# The retromer protein ZmVPS29 regulates maize kernel morphology likely through an auxin‐dependent process(es)

**DOI:** 10.1111/pbi.13267

**Published:** 2019-10-17

**Authors:** Lin Chen, Yong‐Xiang Li, Chunhui Li, Yunsu Shi, Yanchun Song, Dengfeng Zhang, Haiyang Wang, Yu Li, Tianyu Wang

**Affiliations:** ^1^ Institute of Crop Sciences Chinese Academy of Agricultural Sciences Beijing China; ^2^ School of Life Sciences State Key Laboratory for Conservation and Utilization of Subtropical Agro‐Bioresources South China Agricultural University Guangzhou China

**Keywords:** auxin, kernel morphology, maize, retromer, *ZmVPS29*

## Abstract

Kernel size and morphology are two important yield‐determining traits in maize, but their molecular and genetic mechanisms are poorly characterized. Here, we identified a major QTL,*qKM4.08*, which explains approximately 24.20% of the kernel morphology variance in a recombinant population derived from two elite maize inbred lines, Huangzaosi (HZS, round kernel) and LV28 (slender kernel). Positional cloning and transgenic analysis revealed that *qKM4.08* encodes ZmVPS29, a retromer complex component. Compared with the *ZmVPS29* HZS allele, the *ZmVPS29* LV28 allele showed higher expression in developing kernels. Overexpression of *ZmVPS29* conferred a slender kernel morphology and increased the yield per plant in different maize genetic backgrounds. Sequence analysis revealed that *ZmVPS29* has been under purifying selection during maize domestication. Association analyses identified two significant kernel morphology‐associated polymorphic sites in the *ZmVPS29* promoter region that were significantly enriched in modern maize breeding lines. Further study showed that *ZmVPS29* increased auxin accumulation during early kernel development by enhancing auxin biosynthesis and transport and reducing auxin degradation and thereby improved kernel development. Our results suggest that *ZmVPS29* regulates kernel morphology, most likely through an auxin‐dependent process(es).

## Introduction

Maize (*Zea mays* ssp. mays) is one of the most widely cultivated crops worldwide, and its current total grain production has topped that of any other crops (http://www.fao.org/home/en/). The kernel size and shape are two major components of maize yield and have been extensively selected during maize domestication and breeding processes (Liu *et al*., 2017). The wild ancestor of maize, teosinte, has hard glumes and invaginated inflorescence axes, whereas modern maize has naked kernels and flat rachis. During the maize breeding process, kernels of larger size are preferred by breeders due to the positive impact on grain yield (Doebley, 2004; Li *et al*., 2011). The kernel morphology (KM) refers to the ratio of kernel length to kernel width in maize and is a seed‐quality trait that plays an important role in the packaging and acceptability of the seeds for sale to farmers (Pinnisch *et al*., 2012). Based on the KM value, maize kernels can be divided into the following categories: large rounds, large flats, middle rounds, middle flats, small rounds and small flats (Pinnisch *et al*., 2012). Thus, understanding the genetic basis of kernel development might aid further improvements in grain yield and its commodity in maize.

Numerous studies have been devoted to the dissection of the genetic bases regulating maize kernel size and shape. Quantitative trait loci (QTLs) analyses have mapped hundreds of QTLs for kernel‐related traits, including kernel length, kernel width, kernel thickness and hundred‐kernel weight (HKW), using various genetic populations (Chen *et al*., 2016, 2017; Jiang *et al*., 2015; Li *et al*., 2013; Liu *et al*., 2017; Martinez *et al*., 2016; Peng *et al*., 2011; Tang *et al*., 2009). A recent study identified nine QTLs related to KM using a triple testcross population (Jiang *et al*., 2015). However, none of the identified QTLs have been molecularly cloned or functionally characterized. In contrast, recent mutational studies have identified a number of genes that regulate kernel development, such as *SMALL KERNEL 1* (encoding a PPR‐E protein; Li *et al*., 2014), *Supernumerary Aleurone Layers 1* (encoding a vacuolar sorting protein; Shen *et al*., 2003), *U6 Biogenesis‐Like 1* (encoding an RNA exonuclease; Li *et al*., 2017) and *Unhealthy Ribosome Biogenesis 2* (encoding an Urb2 domain‐containing protein; Wang *et al*., 2018). Of particular interest, numerous studies have shown that maize mutants defective in auxin biosynthesis, transport or response display a wide range of abnormalities in the development of the embryo*,* endosperm, spikelet‐pair meristem or branch meristem, kernel number and kernel weight (Barazesh and McSteen, 2008; Bernardi *et al*., 2012; Chen *et al*., 2014; Forestan *et al*., 2010, 2012; Galli *et al*., 2015; Scanlon *et al*., 2002). Additionally, it has been shown that sucrose released from the maternal tissue into the developing kernel is involved in the activation of *ZmYUC* expression and therefore the accumulation of free auxin in the basal endosperm transfer layer cells required for kernel development (LeClere *et al*., 2010; Scanlon *et al*., 2002). More recently, GWAS and transcriptome analyses have been applied to identify candidate genes associated with maize kernel development (Fu *et al*., 2013; Zhang *et al*., 2016). Altogether, these studies have revealed a complex interplay among several phytohormones, including auxin, brassinosteroids and cytokinin, in the regulation of maize kernel development (Doll *et al*., 2017; Jiang *et al*., 2013; Li and Li, 2016; Locascio *et al*., 2014). Despite the progress, our understanding of the genetic control of maize kernel development remains fragmented.

In this study, we identified a major QTL (*qKM4.08*) associated with KM using a recombinant inbred line (RIL) population. We isolated the candidate gene based on linkage mapping and verified its function through expression analysis and genetic transformation assays. We showed that *qKM4.08* encodes a retromer complex subunit, ZmVPS29, that mainly acts to regulate grain width (thus impacting the KM) likely through an auxin‐dependent process(es). Sequence analyses suggest that *ZmVPS29* has been under purifying selection during maize domestication and likely in breeding processes as well. Our results provide valuable targets for future improvement in the maize KM and yield through molecular breeding.

## Results

### Phenotype analysis and QTL mapping for KM

**Figure 1:**
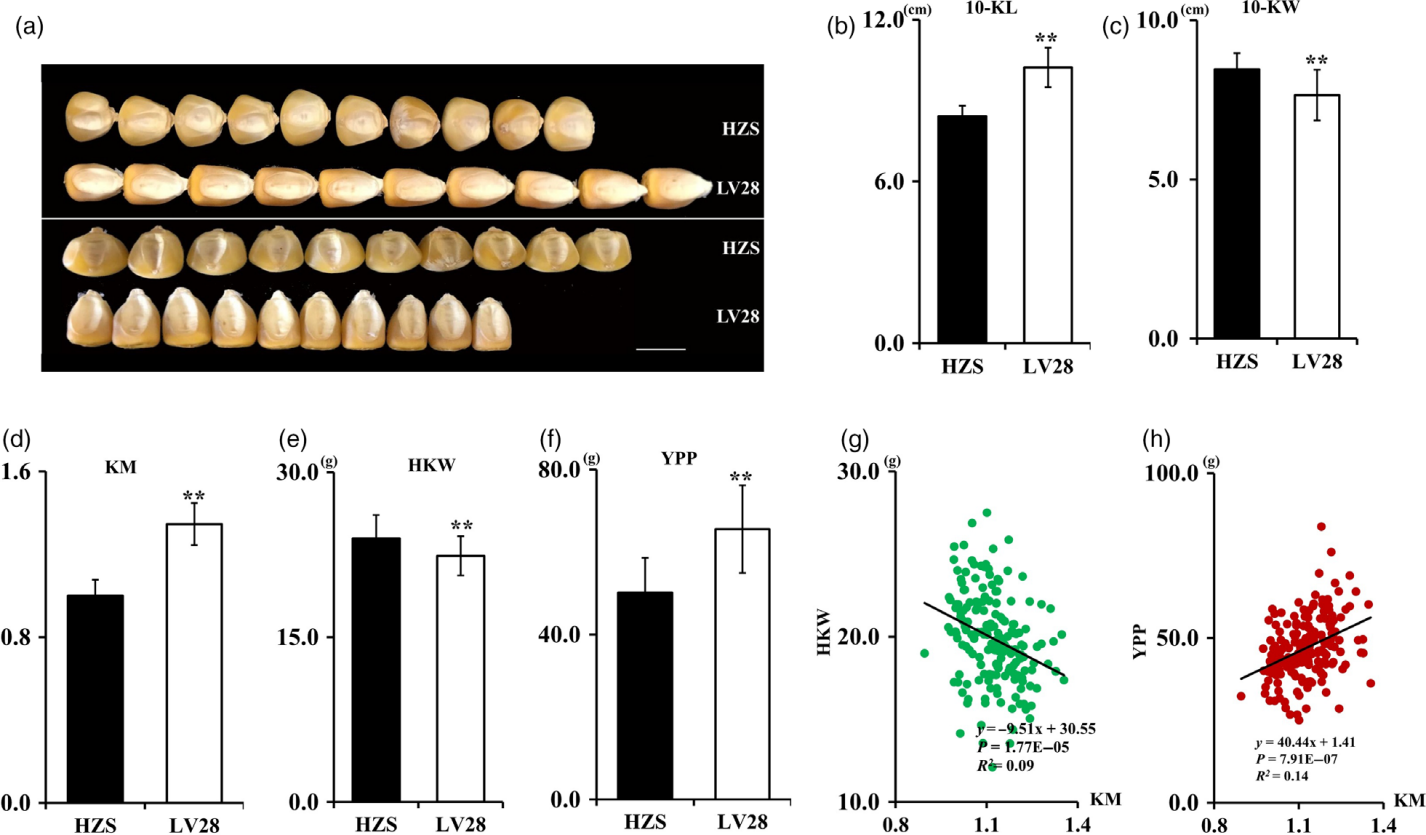
Phenotype analysis. (a) Kernel morphology of Huangzaosi (HZS) and LV28. Bars, 1 cm. (b–f) Statistical data for HZS and LV28 kernel‐related traits, *n* = 35, **P < 0.01 (*t*‐test). (b) Kernel morphology (KM). (c) 10‐Kernel length (10‐KL). (d) 10‐Kernel width (10‐KW). (e) Hundred‐kernel weight (HKW). (f) Yield per plant (YPP). (g) Correlation between the KM value and HKW in the RIL population. (h) Correlation between the KM value and YPP in the RIL population.

To dissect the genetic basis of the maize KM, we conducted a QTL analysis using an RIL population of Huangzaosi (HZS) and LV28 previously generated in our laboratory (Li *et al*., 2013). Both HZS and LV28 are elite inbred lines widely used in Chinese maize breeding programs and are the founder lines for the TSPT (Tang Si Ping Tou) and Ludahonggu heterotic groups, respectively (Wang *et al*., 2008; Wu *et al*., 2014). HZS has a medium round kernel, whereas LV28 has a medium flat kernel (Figures 1a, b). Additionally, these two parental lines also exhibit distinct differences with respect to kernel size and other yield‐related traits, such as 10‐kernel length (10‐KL), 10‐kernel width (10‐KW), HKW and grain yield per plant (YPP) (Figure 1c–f). As previously reported (Pinnisch *et al*., 2012), we also found that HKW and KM were negatively correlated, whereas YPP and KM were positively correlated in this RIL population (Figures 1g, h). The seed coat at 6 DAP was used for cytological observation. The results showed that LV28 had medium flat cells and HZS had round cells. LV28 had longer and narrower cells compared with HZS (Figures S1a, b).

Using this population, we identified 11 and 12 QTLs for 10‐KL and 10‐KW (Li *et al*., 2013), respectively. In this study, nine QTLs for KM were found using this population (Figure S2a, b). Some of these QTLs were related to more than one trait (Figure S2c, Tables S1 and S2). *qKM4.08*, which could explain 24.20% of the phenotypic variance in the KM, was the only major QTL for KM in this population and was constantly found in different environments (Table S1). *qKM4.08* also explained 13.18% of the phenotypic variance in 10‐KL and 8.76% of the phenotypic variance in 10‐KW. Furthermore, linkage analysis using the BC_1_F_1_ population and BC_3_F_1_ backcross populations derived from HZS (as the donor parent) and LV28 (as the recurrent parent) repeatedly mapped *qKM4.08* to a region between umc1940 and umc2287 on chromosome 4 (Figure S2d and Table S3). Thus, we selected *qKM4.08* for further studies.

### Fine‐mapping of *qKM4.08*

**Figure 2:**
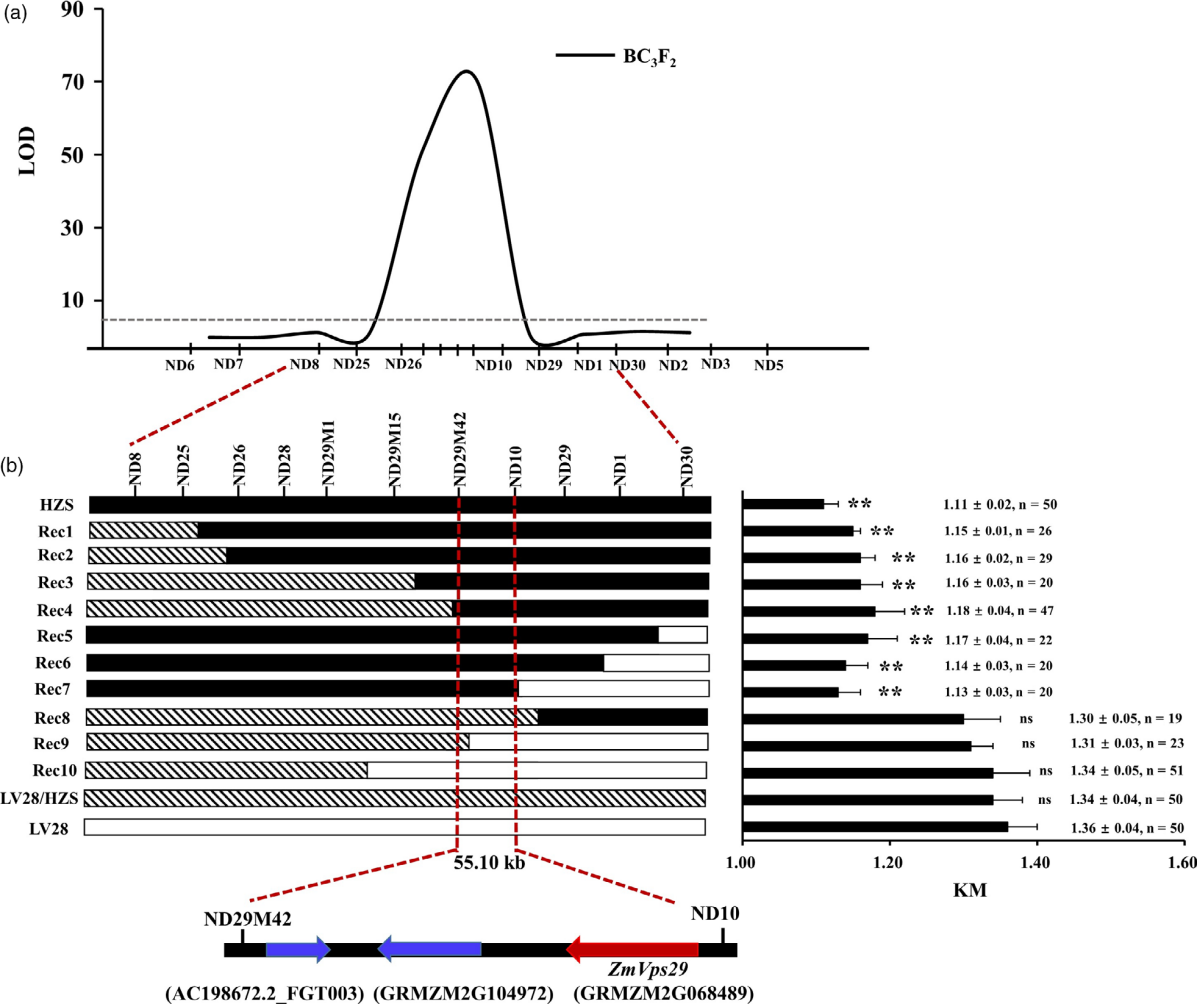
Fine‐mapping of *qKM4.08*. (a) Analysis of QTLs for KM in the BC_3_F_2_ population using a set of indel markers. (b) Genotypes and phenotypes of selected recombinants in the *qKM4.08* region. The number of plants used in the phenotyping analysis is shown on the right. (c) The final mapping region contains the following three genes: AC198672.2_FGT003, GRMZM2G104972 and GRMZM2G068489. GRMZM2G068489 is the candidate gene *ZmVPS29*. ns means not statistically significant; **P < 0.01 (*t‐*test).

To fine‐map *qKM4.08*, we developed a larger BC_3_F_2_ population (with 4521 individuals) and new insertion/deletion polymorphism (indel) markers around *qKM4.08* (Table S4). Additionally, we found that *qKM4.08* could be detected in this population (Figure 2a). A total of 10 recombination events were identified in the *qKM4.08* interval ND8‐ND30. Phenotypic analysis of these recombinants showed that the KM values of Rec1‐Rec7 (which carry the HZS allele in the ND29M42‐ND10 region) were distinctly lower than that of LV28, but no significant differences were found in the KM values of Rec8‐Rec10 (which carry the LV28 allele in the ND29M42‐ND10 region) with LV28 (Figure 2b). We also found that significant differences exist for 10‐KW between LV28 and the recombinant individuals, but no differences could be found for 10‐KL (Figure S3). These results indicated that the ND29M42‐ND10 interval might contain the gene underlying *qKM4.08*, with the LV28 allele for larger KM and the HZS allele for smaller KM.

Sequence analysis based on the published B73 genome (Schnable *et al*., 2009) revealed that the ND29M42‐ND10 interval covers approximately 55.10 kb and harbours only three genes, that is AC198672.2_FGT003, GRMZM2G104972 and GRMZM2G068489 (Figure 2c). Expression analysis showed that only GRMZM2G068489 was expressed in the developing kernels (Figure S4a). Therefore, we speculated that GRMZM2G068489 might be the candidate gene for *qKM4.08*. GRMZM2G068489 is predicted to encode a protein that is highly homologous to Arabidopsis Vacuolar Protein Sorting 29 (AtVPS29) (amino acid identity: 87.37%) (Figure S4b). Hence, we designated GRMZM2G068489 as *ZmVPS29* hereafter. Phylogenetic analysis showed that orthologs of ZmVPS29 are widespread in both monocots and dicots (Figure S4c).

### Overexpression of ZmVPS29 alert kernel morphology

**Figure 3:**
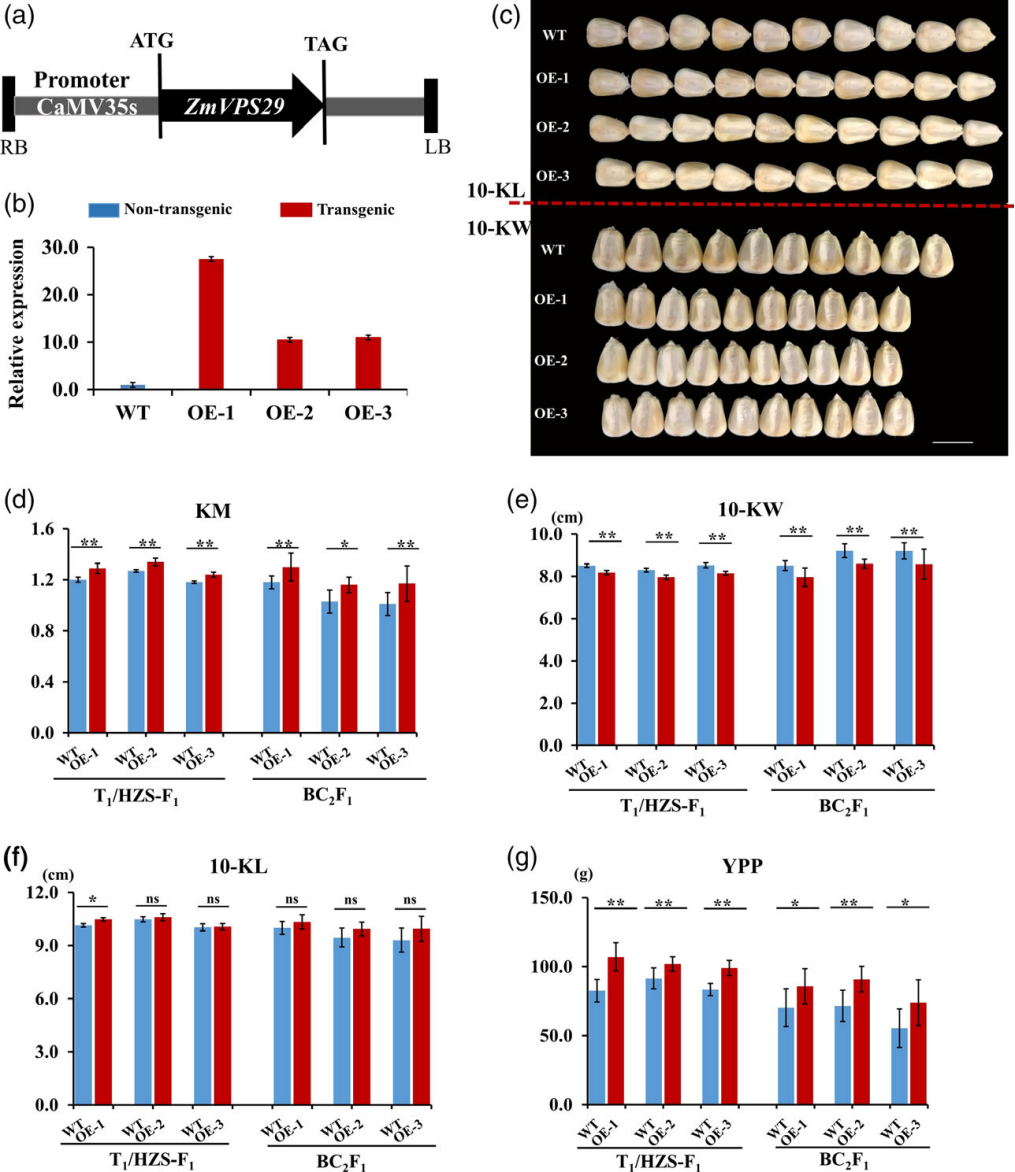
Transgenic validation of *ZmVPS29*. (a) Diagram of the *ZmVPS29* overexpression construct. LB, left border; RB, right border. (b) Quantitative RT‐PCR analysis showing the relative expression levels of *ZmVPS29* in the kernels of T_1_ transgenic plants from three independent transgenic events at 6 DAP. The data were normalized to nontransgenic plant samples. The expression values are presented as the means ± SEs. Each sample contains five individuals. (c) Kernel characteristics of the *ZmVPS29* overexpression plants versus nontransgenic plants. Bars, 1 cm. (d–g) Comparison of kernel size and yield‐related traits between the *ZmVPS29*‐overexpressing plants and nontransgenic plants in the T_1_/HZS‐F_1_ and corresponding BC_2_F_1_ populations (three independent transgenic events were used in this analysis). (d) KM (10‐KL/10‐KW). (e) 10‐Kernel width (10‐KW). (f) 10‐Kernel length (10‐KL). (g) Yield per plant (YPP). The values are presented as the means ± SDs. ns means no significant difference, *P < 0.05, **P < 0.01 (*t*‐test).

To verify the function of ZmVPS29 in regulating KM, we generated several *ZmVPS29* overexpression lines (driven by the CaMV35S promoter) (Figure 3a). Three independent transgenic events were obtained by introducing *ZmVPS29* into the maize hybrid Hi‐II (Figure 3b). Next, we constructed the T_1_/HZS‐F_1_ and BC_2_F_1_ populations by crossing the transgenic T_1_ plants with HZS to evaluate the effects of *ZmVPS29* on KM values. We found that the *ZmVPS29* overexpression transgenic plants had significantly reduced kernel widths, but not kernel lengths, compared to the nontransgenic control plants (Figures 3c–f and Table S5). No obvious differences in HKW or kernel row number (KRN) were found between the transgenic and nontransgenic plants (Figures S5a, b). However, the transgenic plants also showed increases in the YPP (Figure 3g) and kernel number per row (KNPR) (Figure S5c). Similar results were obtained using the T_1_/Z58‐F_1_ and BC_2_F_1_ populations constructed in the Zheng58 background (Z58, another elite maize inbred line widely used in maize breeding in China) (Figure S6a–g and Table S6). Furthermore, a correlation analysis performed using the T_1_/HZS‐F_1_ and BC_2_F_1_ populations in the HZS background revealed a significant positive correlation between KM and YPP (Figure S7a–f). These results collectively suggest that *ZmVPS29* overexpression can confer slenderer kernels (mainly due to reduced kernel width), a higher KNPR and a higher YPP.

### Expression pattern of *ZmVPS29*

**Figure 4:**
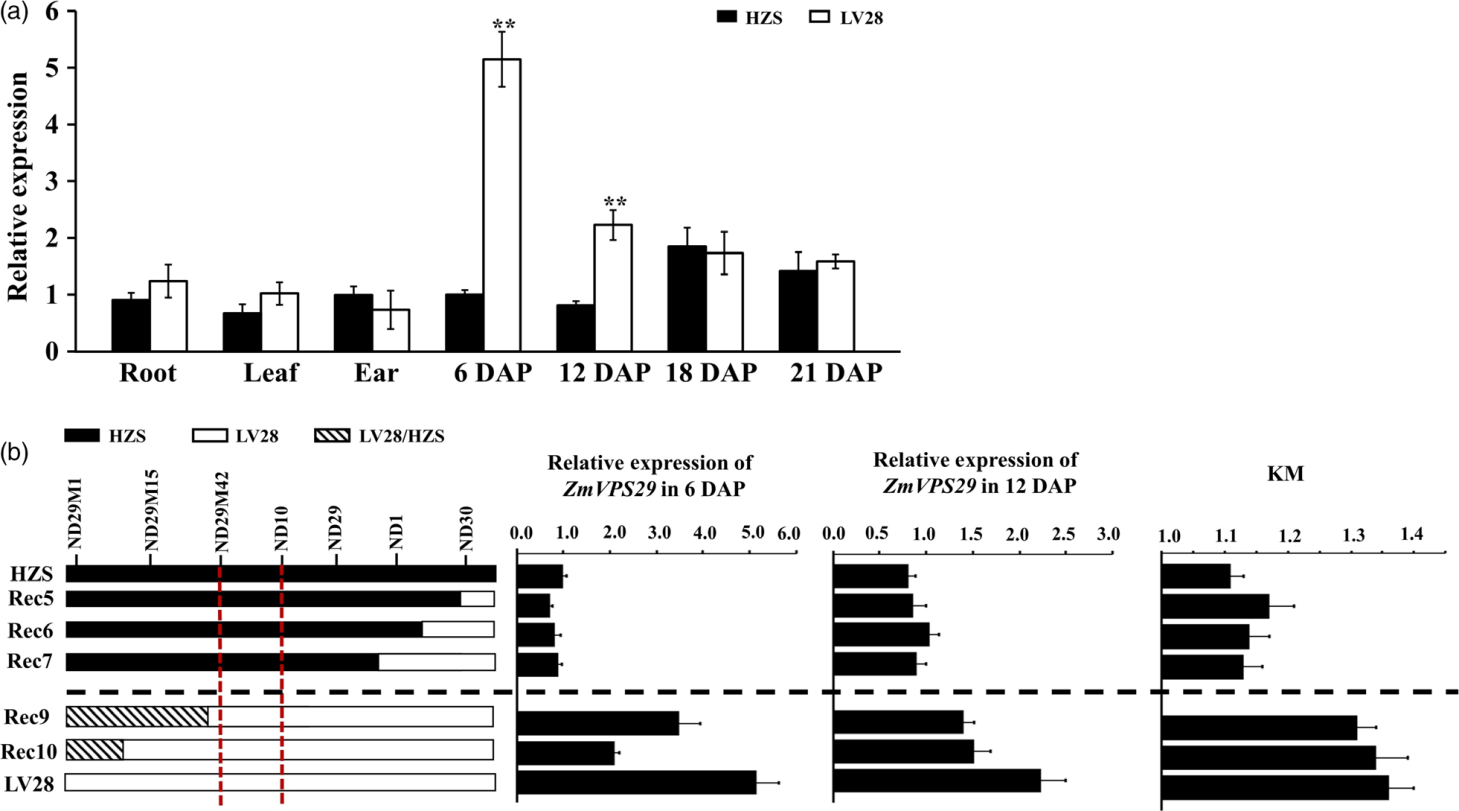
Expression pattern of *ZmVPS29*. (a) Quantitative RT‐PCR analysis of *ZmVPS29* expression in HZS and LV28. The relative expression levels of *ZmVPS29* in the root, leaf, ear and kernel at different developmental stages are shown. The expression levels of *ZmVPS29* were normalized to those of *GAPDH* (NM_001111943). Three biological replicates were used for each sample. The values are presented as the means ± SDs. **P < 0.01 (*t*‐test). (b) Expression analysis of *ZmVPS29* in kernels from various recombinant lines at 6 and 12 DAP. The white, black and striped boxes in the graph denote genomic segments derived from the LV28, HZS and heterozygous regions, respectively.

A real‐time quantitative reverse transcriptase (RT)‐PCR analysis showed that *ZmVPS29* was expressed in various organs, including the root, leaf, ear and developing kernels, and the relative expression levels of *ZmVPS29* at 6 DAP were significantly higher in kernels of LV28 than in those of HZS (Figure 4a). Consistent with this finding, we found that Rec5, Rec6 and Rec7, which carry the HZS allele in the *qKM4.08* region, showed lower expression of *ZmVPS29* and lower KM values than those of Rec9 and Rec10, which carry the LV28 allele in the *qKM4.08* region (Figure 4b).

**Figure 5:**
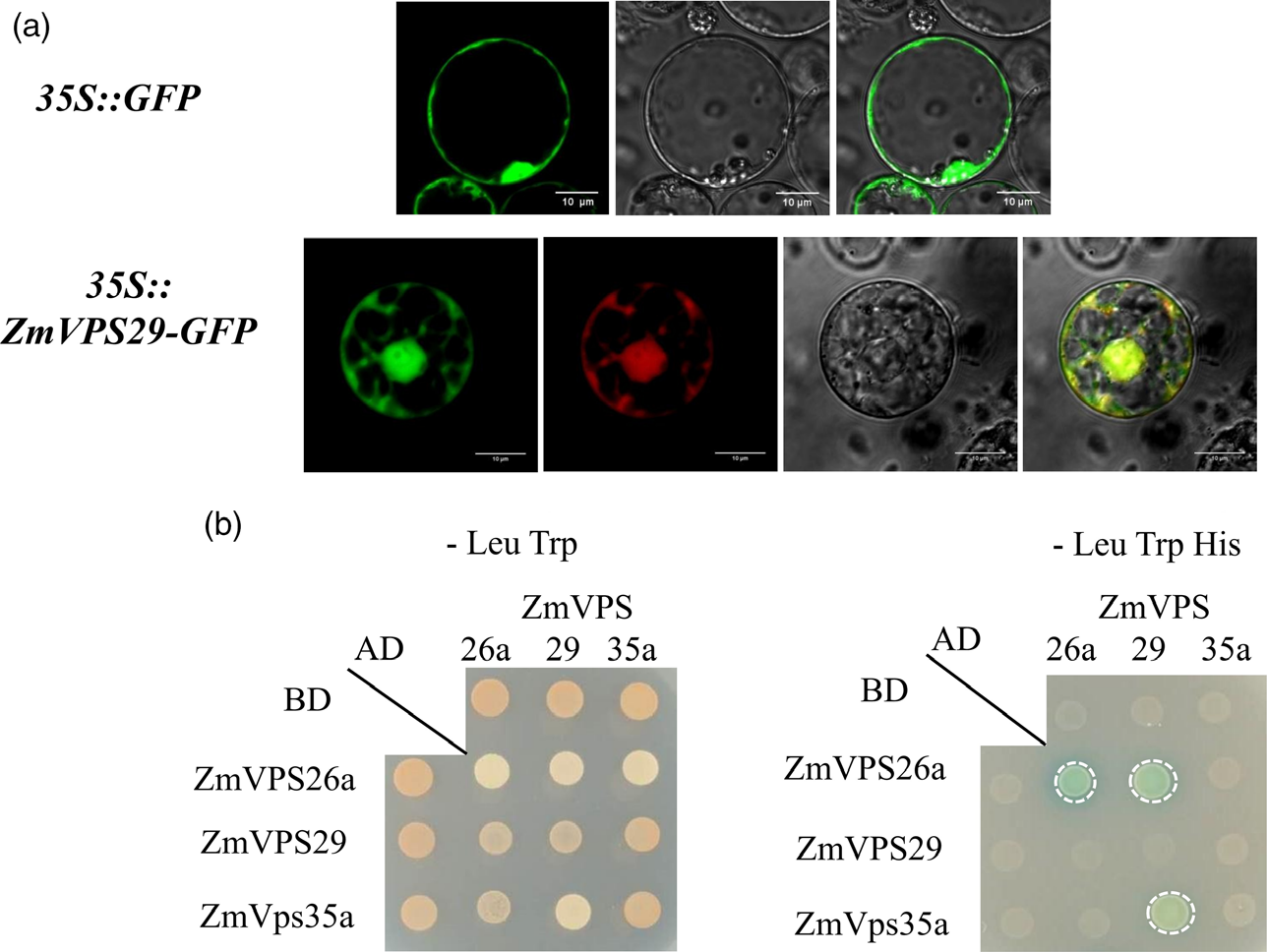
ZmVPS29 interacts with other plant retromer proteins. (a) Subcellular localization of ZmVPS29. The first line is CaMV35S:GFP, which was used as a control. The second line is the CaMV35S:ZmVPS29‐GFP construct, which was used to assess protein localization, and the marker is the mKate protein. Confocal observation of maize protoplasts (scale bar, 10 μm). (b) A yeast two‐hybrid assay shows that ZmVPS29 interacts with ZmVPS35a (GRMZM5G825524) and ZmVPS26a (GRMZM2G136563), two other components in the VPS35‐VPS26‐VPS29 retromer subcomplex. AD refers to activation domain, and BD means DNA‐binding domain. The dashed circle suggests positive interactions.

Sequence analysis indicated that the protein sequences encoded by the HZS allele and LV28 allele are identical (Figure S8). To determine the subcellular localization of ZmVPS29, we constructed a ZmVPS29‐GFP fusion protein construct (driven by the CaM35S promoter) and transiently expressed it in maize protoplasts. Similar to the free GFP control, ZmVPS29‐GFP was widely expressed in the cytomembrane, nucleus and cytoplasm (Figure 5a). Previous studies have suggested that VPS29 is a component of the Vps35/Vps29/Vps26 retromer subcomplex in yeast and *Arabidopsis* (Jaillais *et al*., 2007; Shimada *et al*., 2006). Thus, we investigated whether this retromer subcomplex also exists in maize. A two‐hybrid assay confirmed the physical interaction between ZmVPS29 with two other components in the retromer subcomplex, ZmVPS26a and ZmVPS35a (Figure 5b), which suggests that ZmVPS29 acts as a conserved component in the retromer complex in maize.

### Association and evolution analysis of *ZmVPS29*

**Figure 6:**
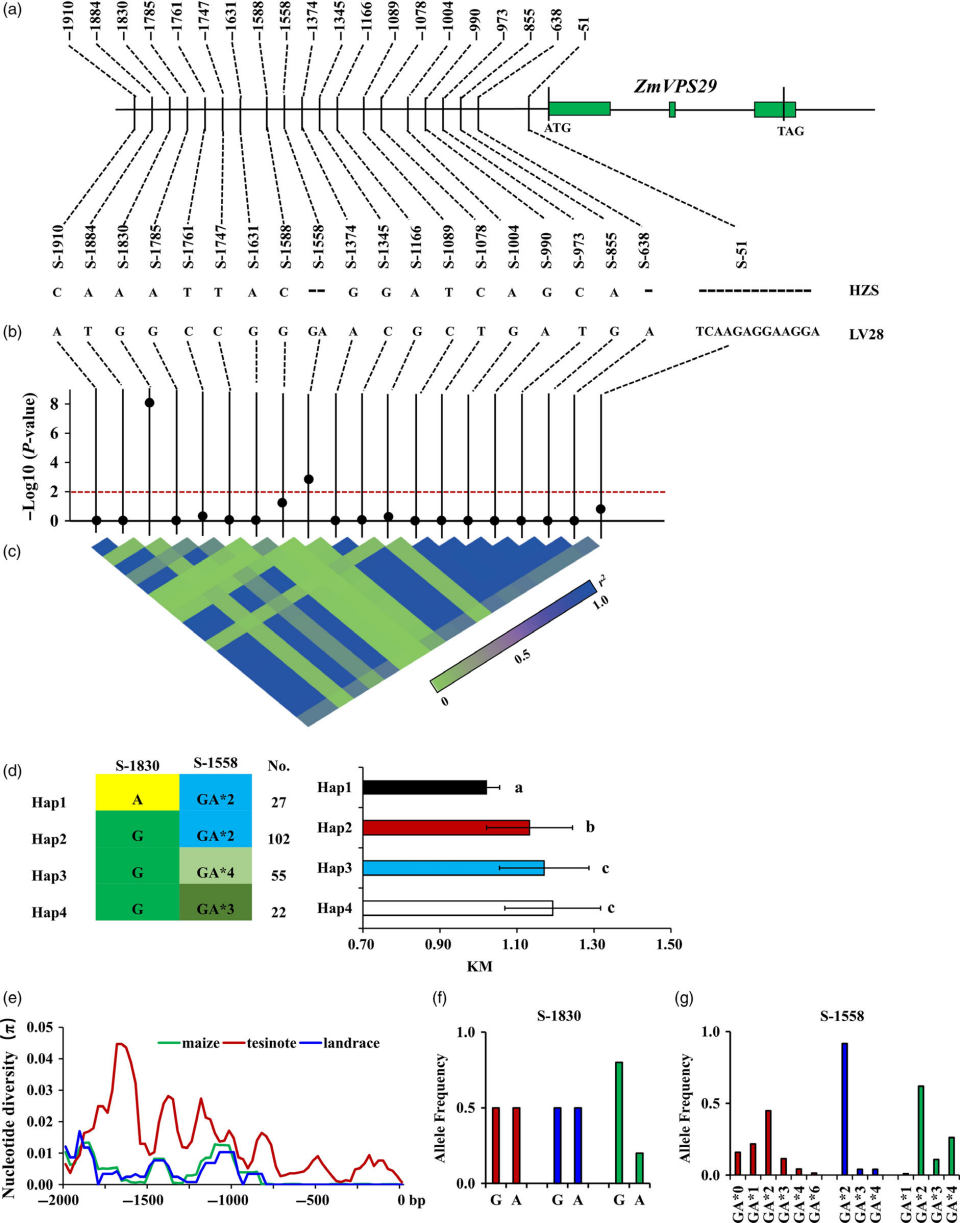
Association and evolutionary analysis of the promoter region of *ZmVPS29*. (a) Sequence comparison of the promoter region between HZS and LV28. (b) Association analysis for the KM value with the 20 variants in the promoter region of *ZmVPS29*. Black dots represent the 20 variations. (c) Triangle matrix of the pairwise linkage disequilibrium. (d) Comparison of different haplotypes. Four haplotypes could be identified based on the two polymorphic sites (S‐1830 and S‐1558) in the association panel. HZS belongs to the Hap1 group, whereas LV28 belongs to the Hap4 group. Significant differences were found among these haplotype groups. No. indicates the number of lines belonging to the corresponding haplotype group, and ns means no significant difference, *P < 0.05, **P < 0.01 (*t*‐test). (e) Nucleotide diversities (π) within the ~2‐kb promoter region of *ZmVPS29* for maize inbred lines, landraces and teosinte accessions. (f) Allele frequencies of S‐1830 in the teosinte accessions, landraces and maize inbred lines. (g) Allele frequencies of S‐1558 in the teosinte accessions, landraces and maize inbred lines. The *x*‐axis indicates the number of accessions for the ‘GA’ allele.

The higher expression of the LV28 allele and the identical amino acid sequences of the HZS and LV28 alleles suggest that the causal variations might lie in their regulatory sequences. Sequence analysis of the promoter region (1954 bp upstream of the start codon of *ZmVPS29*) identified three indels and 17 single nucleotide polymorphisms (SNPs) between the HZS and LV28 alleles (Figures 6a and S9). To identify the causative sites responsible for the KM differences between HZS and LV28, we examined the allelic variations of the 20 sequence polymorphisms in 210 inbred maize lines (Table S7). The results showed that two polymorphic sites in the promoter region of *ZmVPS29*, S‐1830 and S‐1558 were significantly associated with KM value variations at the *P* < 0.01 level (Figure 6b, c and Tables S8 and S9). Furthermore, we identified four haplotypes for these 201 maize inbred lines based on these two polymorphic sites. Phenotypic analysis showed that Hap1 (to which HZS belongs) had the lowest KM value (characterized as round kernels) and Hap4 (to which LV28 belongs) had the highest KM value (characterized as slender kernels) (Figure 6d). Interestingly, there was a significant difference for 10‐KW, but not for 10‐KL, between the Hap1 group and Hap4 group (Figures S10a, b), which indicates that *ZmVPS29* mainly affects KW.

To trace the evolutionary history of *ZmVPS29* during maize domestication and breeding, we sequenced the promoter regions of the *ZmVPS29* gene from 59 teosinte and 30 landrace accessions and compared their nucleotide diversity (π) values with those of the maize inbred lines. We found that the promoter region of *ZmVPS29* had undergone a strong reduction in nucleotide diversity from teosinte to the landrace and the maize inbred lines, with π_maize_/π_teosinte_ = 0.29, which suggested that only 29% of the nucleotide diversity found in the teosinte accessions was retained in the maize inbred lines (Figure 6e). Additionally, the nucleotide diversity of landrace and maize inbred lines was very similar (π_maize_ =0.0036_,_ π_landrace_ =0.0033).

Furthermore, an analysis of the allele frequency showed that the ‘G’ nucleotide at S‐1830 had a higher frequency (0.80) in the maize inbred lines than in the teosinte accessions (0.50), which implied that the ‘G’ nucleotide at S‐1830 is a standing variation in teosinte and has undergone purifying selection during maize breeding process for its slender KM (Figure 6f). Additionally, the frequencies of the ‘GAGA’ and ‘GAGAGAGA’ types at S‐1558 were also significantly increased in modern maize, and this effect was accompanied by a reduction in other ‘GA’ insertion types at this locus (Figure 6g).

### *ZmVPS29* regulates KM likely through an auxin‐dependent process

**Figure 7:**
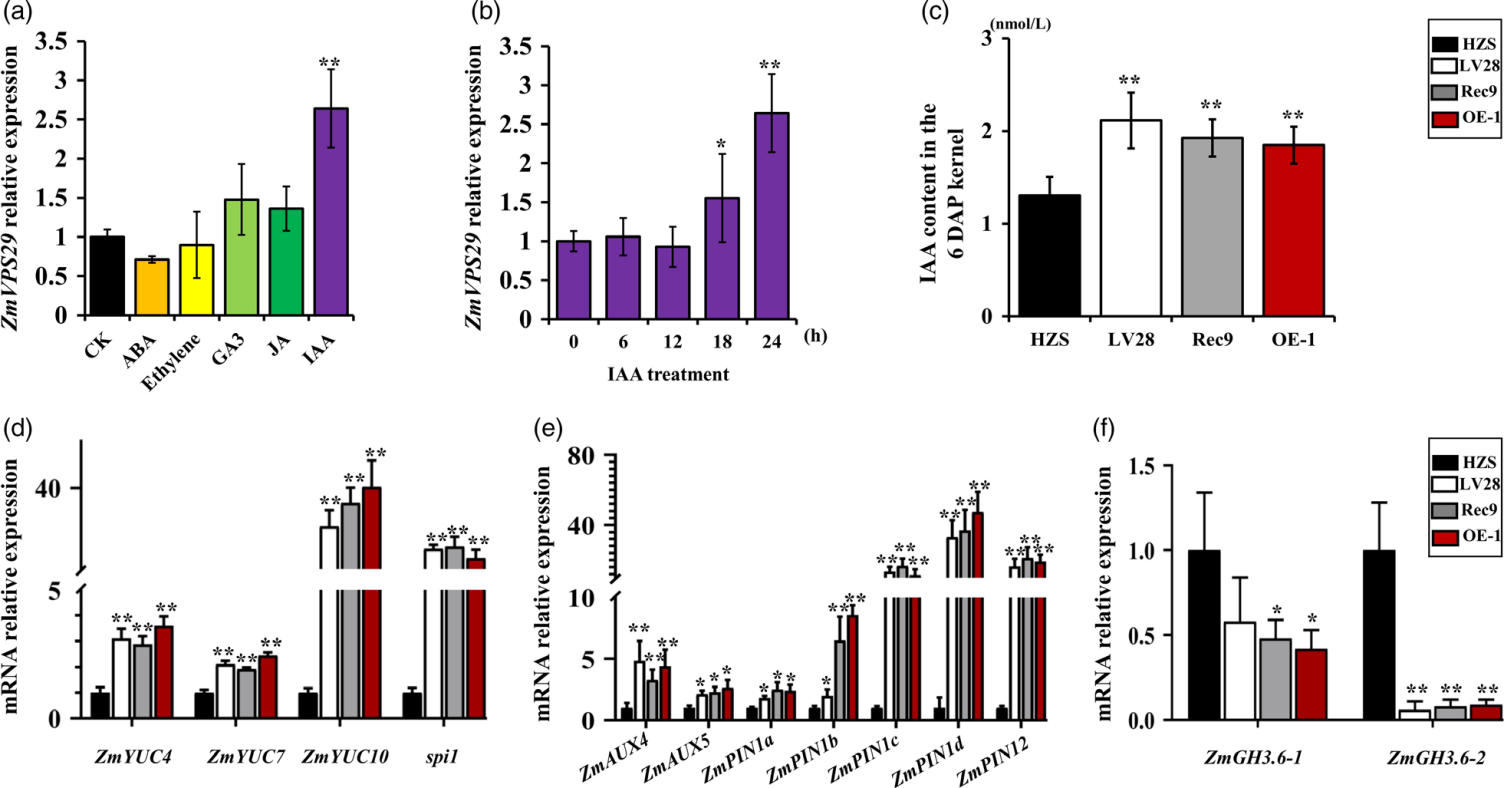
ZmVPS29 increases auxin accumulation in kernels at 6 DAP via coordinated auxin biosynthesis, transport and degradation. (a) Relative expression levels of *ZmVPS29* subjected to treatments with different phytohormones. CK, check experiment (B73); GA3, gibberellic acid; IAA, indole‐3‐acetic acid; JA, jasmonic acid. A qRT‐PCR analysis shows that expression of *ZmVPS29* is specifically regulated by IAA and not by other hormones. (b) Time‐course response of *ZmVPS29* to 10 μM IAA treatment. The values are presented as the means ± SDs (*n* = 3). (c) Quantification of auxin content in the kernels at 6 DAP. (d–f) Relative transcription levels of the genes involved in auxin biosynthesis, transport and degradation.

Previous studies have indicated that AtVPS29 is involved in auxin processes. A sequence analysis showed the presence of three AuxREs (auxin response elements) in the promoter region of *ZmVPS29* and indicated that ZmVPS29 is involved in auxin processes in maize (Figure S9). To further investigate the molecular basis of ZmVPS29 in regulating KM, we tested the effects of various phytohormones on the expression of *ZmVPS29*. qRT‐PCR analysis showed that *ZmVPS29* was specifically induced by treatment with exogenous auxin, but not by other hormones (Figure 7a, b). The endogenous auxin contents in the kernels from different maize genotypes at 6 DAP were observed. As shown in Figure 7c, the content of free IAA was increased markedly at 6 DAP in kernels from LV28‐, Rec9‐ and ZmVPS29‐overexpressing plants. The endogenous auxin accumulation is evidently determined by a combination of auxin biosynthesis, transport and degradation. To clarify whether *ZmVPS29* affects the auxin levels by regulating these processes, we examined the relative expression levels of a number of auxin biosynthesis genes, such as *ZmYUC*s, and polar auxin transport genes, such as *ZmPIN*s, in the maize kernel at 6 DAP. As expected, we found that *ZmYUC4*,* ZmYUC7*,* ZmYUC10* and *spi1* are more highly expressed in LV28, Rec9 and OE‐1 than in HZS (Figure 7d). As to *ZmAUX*s and *ZmPIN*s, the expression levels of *ZmAUX4*,* ZmAUX5*,* ZmPIN1a*,* ZmPIN1b*,* ZmPIN1c*,* ZmPIN1d* and *ZmPIN12* were significantly increased in LV28, Rec9 and OE‐1 plants. In addition, genes involved in auxin degradation processes, such as *ZmGH3.6‐1* and *ZmGH3.6‐2*, were substantially reduced in LV28, Rec9 and OE‐1 plants (Figure 7e). Other genes involved in auxin biosynthesis, transport and degradation did not show significant differences in mRNA levels (Figure S11). These data suggest that *ZmVPS29* might play a role in the maintenance of auxin homeostasis *in vivo* by regulating auxin accumulation in maize kernel development through a combination of auxin biosynthesis, transport and degradation.

## Discussion

Maize kernels exhibit a wide range of natural variations in several kernel morphology‐related traits, including kernel length, kernel width, kernel thickness and hundred‐kernel weight (HKW); however, the genetic control underlying these genetic variations is still poorly understood. In this study, we report cloning and characterization of the first major QTL controlling kernel morphology using an RIL population of two elite maize inbred lines that are widely used in China, HZS (round kernel) and LV28 (slender kernel). *qKM4.08* explained approximately 24.20% of the phenotypic variance in the KM, 13.18% in the 10‐KL and 8.76% in the 10‐KW. Molecular cloning revealed that *qKM4.08* encodes ZmVPS29, a retromer complex component. Retromer is a highly conserved protein complex mediating protein trafficking in eukaryotes and is composed of the following two important subcomplexes: a key trimer subcomplex containing three vacuolar protein sorting proteins, VPS35/VPS29/VPS26, and another subcomplex related to sorting nexin proteins (Attar and Cullen, 2010; Nodzyński *et al*., 2013; Seaman, 2005; Wassmer *et al*., 2009). Our yeast hybrid assay confirmed physical interactions between ZmVPS29 with ZmVPS35a and ZmVPS26a (Figure 5b), the other two components of the retromer complex, which suggests that ZmVPS29 likely acts as a conserved component in the retromer complex in maize.

Auxin is an important plant hormone that is essential for many biological processes, and its accumulation is necessary for kernel development in maize (Doll *et al*., 2017). In this study, we showed that *ZmVPS29* appears to promote kernel development by regulating auxin levels in the early kernel. First, ZmVPS29 transcription was positively correlated with KM, a process that requires auxin‐mediated establishment. Second, exogenous auxin application can change the expression of ZmVPS29. Finally, changes in the auxin levels at 6 DAP in the kernels of the ZmVPS29‐overexpressing and introgression lines were clearly confirmed by measurements of the endogenous auxin content. Based on these results, we hypothesize that *ZmVPS29* influences auxin biosynthesis and transport in early kernel development in maize. Previous studies have suggested that *YUC*s are key auxin biosynthesis genes in rice and maize. Higher expression of *YUC*s leads to the overproduction of auxin, and *yuc* mutants display development defects in kernel. Mutations in *ZmYUC1* cause abnormal endosperm development and a reduction in kernel weight (Scanlon *et al*., 2002). Additionally, numerous previous studies have accumulated substantial evidence to support a critical role of auxin in regulating kernel development in maize. For example, mutations in *ZmPIN1*s (*ZmPIN1a* and *ZmPIN1b*)*,* which encode auxin efflux carrier proteins, cause abnormal embryos (Chen *et al*., 2014; Forestan *et al*., 2010, 2012). As a group of early auxin‐responsive genes, the *GH3* family encodes IAA‐amido synthetases that prevent free IAA accumulation, and some members of the *GH3* family in rice possess IAA‐amido synthetase activity. In our study, we found that ZmVPS29 changed the transcription of ZmYUCs, ZmPINs/ZmAUXs and ZmGH3s, and these changes could be one of the reasons explaining the changes in the auxin contents. Therefore, our results strongly suggest that ZmVPS29 regulates kernel development in maize by enhancing auxin accumulation during early kernel development (Figures S12 and S13).

Interestingly, we found that the gene products encoded by the LV28 and HZS alleles of *ZmVPS29* are identical, but the LV28 allele showed higher expression in developing kernels than the HZS allele, which suggests that natural variations in the promoter regions of the LV28 and HZS alleles of *ZmVPS29* might be responsible for the differential expression and functionality of *ZmVPS29* in regulating maize kernel morphology. Consistent with this notion, *ZmVPS29* overexpression also conferred a slender kernel morphology in different maize genetic backgrounds. Additionally, association analyses identified two significant kernel morphology‐associated polymorphic sites (S‐1830 and S‐1558) in the *ZmVPS29* promoter region (Figure 6b). Moreover, our sequence analysis showed that the nucleotide diversity (π) values of *ZmVPS29* in modern maize elite lines were notably lower than those in teosinte (Figure 6e), which suggests that positive selection occurred during maize domestication and breeding. Furthermore, analysis of the allele frequency showed that the ‘G’ nucleotide at S‐1830 had a higher frequency (80%) in maize inbred lines than in the teosinte accessions (50%) (Figure 6f), which suggests that this SNP might represent a causal variation in *ZmVPS29*. Because larger kernels with a slender morphology are preferred in maize breeding, the functional polymorphic sites in the *ZmVPS29* promoter can be introgressed into elite lines with a round KM through molecular marker‐assisted breeding. Alternatively, newly developed CRISPR/Cas9 technologies offer a viable option to modulate the expression pattern and/or levels of *ZmVPS29* through the targeted modification of regulatory elements in the *ZmVPS29* promoter, which would help create an ideal KM that can be stacked with other yield‐related traits to increase maize yield.

## Experimental procedures

### QTL analysis of KM

The RIL population used in this study was derived from a cross between HZS and LV28, both of which are elite inbred lines widely used in Chinese maize breeding. The genotypes and phenotypes of the RIL population have been described previously (Li *et al*., 2013). In this study, we used the KM (the ratio of 10‐KL and 10‐KW) as a single trait for QTL analysis. QTL mapping for KM was performed using the QTL IciMapping software version 4.1 (http://www.isbreeding.net), which is based on the inclusive composite interval mapping (ICIM) algorithm. The logarithm of odds (LOD) threshold was calculated using 1000 permutations at a significance level of *P* = 0.05 (LOD > 2.5), with a scanning interval of 1 cM between markers and a putative QTL. The name of the QTL, *q* + trait abbreviation + chromosome number + QTL genetic position (IBM2008 genetic map), for example *qKM4.08*, corresponds to the QTL for kernel morphology on chromosome 4 and its genetic position in the IBM2008 genetic map bin4.08. Additionally, the interaction between a QTL and environments was analysed by using the QTL IciMapping software version 4.1, with an LOD > 3.0 representing a significant interaction between a QTL and environments.

### Fine‐mapping of *qKM4.08*

The backcross populations were developed using HZS as the recurrent parent. For the BC_1_F_1_ and BC_3_F_1_ populations, simple sequence repeat (SSR) markers (http://www.maizegdb.org) were used in genotyping. According to the results of QTL mapping based on the two backcross populations, 4521 BC_3_F_2_ individuals were derived from individuals who are heterozygous in the *qKM4.08* region. Based on the resequencing information for HZS and LV28 provided by Jinsheng Lai from China Agricultural University, we designed indel or SNP markers with a product size < 300 bp in the *qKM4.08* region interval using Primer Premier 5.0 (Premier Biosoft International, USA). The significance of the phenotypic differences in the different recombinant types relative to LV28 was evaluated using t‐tests in SAS (SAS Institute, Inc., Cary, NC). All the populations used for the fine‐mapping of *qKM4.08* were planted at the Changping Experimental Station of ICS/CAAS in Beijing (39.48° N, 116.28° E) in 2013 and 2014. After harvest, the kernels were threshed from the middle part of the ears to measure the 10‐KW (cm) and 10‐KL (cm) and calculate the KM value. The detailed process for fine‐mapping *qKM4.08* has been created in Figure S12.

### cDNA cloning and sequence analysis

The full‐length cDNA sequence of *ZmVPS29* was amplified using high‐fidelity LA Taq Mix (Takara, http://www.clontech.com/takara). The purified PCR products were cloned into the pLB vector (Tiangen, http://www.tiangen.com) according to the manufacturer's instructions, and three positive clones were sequenced for each sample. Sequence alignment was performed using DNAMAN version 5.2.2 (Lynnon Biosoft, http://www.lynnon.com).

### Expression analysis of *ZmVPS29*

The total RNAs in different tissues were extracted using the TRIzol reagent (Life Technologies, Invitrogen, Carlsbad, CA). Genomic DNA was removed from the total RNA using DNase I (Takara, http://www.clontech.com/takara) treatment. First‐strand cDNA was synthesized by an oligo (dT) primer and M‐MLV reverse transcriptase (Invitrogen). Quantitative PCR experiments were performed using a SYBR Green RT‐PCR Kit and gene‐specific primers. The expression levels were normalized using GAPDH (NM_001111943) as an endogenous control.

### Subcellular localization of ZmVPS29

The *ZmVPS29* cDNA sequence was cloned in frame with pBWA(V)HS‐OSGFP under the control of the CaMV35S promoter. The B73 seeds were placed in an incubator at 25 degrees and then cultured for 7‐10 days under darkness. The CaMV35S:GFP vector and the recombinant CaMV35S:ZmVPS29‐GFP vector with the marker mKate were then transfected into maize protoplasts and examined by using the Olympus FluoView FV1000 confocal microscope software (Olympus, Tokyo, Japan).

### Plant transformation

The full‐length coding sequence of *ZmVPS29* was cloned into the pCAMBIA3301‐CaMV35S vector to generate the pCAMBIA3301‐CaMV35S:*ZmVPS29* overexpression construct. The construct was transformed into the maize hybrid Hi‐II by *Agrobacterium*‐mediated transformation following a previously established protocol (Frame *et al*., 2011). We crossed T_1_ transgenic plants with HZS and Z58 to produce two separate F_1_ populations. In the F_1_ populations, transgenic and nontransgenic individuals were identified using transgene‐specific primers. Additionally, the corresponding BC_2_F_1_ populations were obtained by crossing the transgenic lines with HZS and Z58. A *t*‐test method similar to that described in a previous study (Zuo *et al*., 2015) was used to determine the effect of the transgene on the phenotype.

### Association mapping and molecular evolutionary analysis

The phenotypes and genotypes of the association panel have been described in a previous study (Wu *et al*., 2014). The promoter regions of *ZmVPS29* in the maize inbred lines, the landraces and the teosinte accessions were amplified using gene‐specific primers and high‐fidelity LA Taq Mix (Takara, http://www.clontech.com/takara). The PCR products for each sample were then cloned into the pLB vector (Tiangen, http://www.tiangen.com), and at least three clones were sequenced. The sites with allelic frequencies > 0.05 were used for association analysis. Association mapping was performed with TASSEL 2.1 using an MLM Q+K model (Zhang *et al*., 2010). The population structure and kinship of the panel have been analyzed in a previous study (Wu *et al*., 2014). A total of 1015 SNPs with high genetic diversity and even distribution were used to analyse the population structure via the model‐based approach in the Structure V2.3.3 software. The relative kinship was performed using the TASSEL3.0 package with 41819 SNPs with MAF > 0.05 and missing data < 20%. Haplotypes with a sample size > 2 lines (frequencies > 0.01) were selected for analysis in the association panel. The values of π were calculated using DnaSP version 5.0.

### Yeast two‐hybrid assay

Yeast two‐hybrid assays were performed as previously described (Jaillais *et al*., 2007). Full‐length *ZmVPS29*,* ZmVPS35a* and *ZmVPS26a* were cloned from total cDNAs of leaves from the maize inbred line B73. The cDNAs of the three genes were cloned into the pGAD‐T7 and pGBK‐T7 vectors (Clontech, Mountain View, CA, http://www.clontech.com/). These plasmids were cotransformed as the prey and bait vectors into the Y2H Gold yeast reporter strain and cultured until the OD600 reached 0.5. Different yeast strains were grown on SD/‐Leu/‐Trp plate and SD/‐Leu/‐Trp/‐Ade/‐His plates.

### Hormone treatment and IAA measurement

For the analysis of *ZmVPS29* expression after different hormone treatments, 15‐day‐old B73 plants were immersed in liquid 1/2 MS medium containing different hormones (10 μM ABA, ethylene, gibberellin or IAA). The leaves from five plants were used for each treatment and collected for RNA isolation. Mock‐treated B73 plants were used as the control. Kernels from the different maize genotypes (HZS, LV28, Rec9 and OE‐1) at 6 DAP were collected for IAA content analysis. IAA extraction and content measurements were conducted according to previous reports using a UPLC‐MS/MS system (Fu *et al*., 2012).

## Conflicts of interest

The authors declare no conflict of interests.

## Author contributions

H. Wang, Y. Li and T. Wang designed the project. L. Chen and Y‐x. Li conducted the experiments. C. Li, Y. Shi and Y. Song participated in some experiments. L. Chen analysed the data and wrote the manuscript. H. Wang, Y. Li and T. Wang revised the manuscript. All the authors read and approved the manuscript. No conflict of interests are declared.

## Supporting information


**Figure S1** Morphology differences at 6 DAP between HZS and LV28 at the cytological level.
**Figure S2** QTL analysis for KM.
**Figure S3** Genotypes and phenotypes (10‐KL and 10‐KW) of the selected recombinants in the *qKM4.08* region.
**Figure S4** (a) Expression analysis of the three genes contained in the final mapping region of *qKM4.08*. M indicates the molecular marker. (b) Amino acid sequence alignment of the AtVps29 and ZmVPS29 proteins. (c) Phylogenetic tree of ZmVPS29 and related proteins.
**Figure S5** Comparison of yield‐related traits in the T_1_/HZS‐F_1_ and corresponding BC_2_F_1_ populations.
**Figure S6** Transgenic validation of *ZmVPS29* in the Zheng58 (Z58) background.
**Figure S7** Correlation between KM value and kernel yield per plant (YPP).
**Figure S8** Alignment of the nucleotide sequences of the *ZmVPS29* coding region in HZS and LV28.
**Figure S9** Alignment of the nucleotide sequences of the *ZmVPS29* promoter region in HZS and LV28.
**Figure S10** Comparison of 10‐KL (a) and 10‐KW (b) in the four haplotype groups. The values are presented as the means ± SDs.
**Figure S11** Expression of genes associated with auxin biosynthesis, transport, and degradation.
**Figure S12** A putative model depicting the role of ZmVPS29 in the regulation of kernel development in maize.
**Figure S13** Process for the fine mapping of *qKM4.08*.

**Table S1** Summary of QTLs for kernel length, kernel width and kernel morphological in the RIL population.
**Table S2** Quantitative trait locus correspondence likelihood expected by chance between the three kernel traits.
**Table S3** Validation of the effects of *qKM4.08* in different populations.
**Table S4** Primers used in this study.
**Table S5** Phenotypes of the transgenic plants in the HZS background.
**Table S6** Phenotypes of the transgenic plants in the Z58 background.
**Table S7** Association panel used in this study.
**Table S8** Association between kernel size and polymorphism sites in *ZmVPS29*.
**Table S9** Information on the landraces and teosinte accessions used in this study.
