# Supplementary material for: The retromer protein ZmVPS29 regulates maize kernel morphology likely through an auxin‐dependent process(es)
Source: Plant Biotechnol J. 2019 Oct 17;18(4):1004–14. doi: 10.1111/pbi.13267 (PMC7061865; doi:10.1111/pbi.13267)
Supplement: Supplementary file 1 — Figure S1 Morphology differences at 6 DAP between HZS and LV28 at the cytological level. Figure S2 QTL analysis for KM. Figure S3 Genotypes and phenotypes (10‐KL and 10‐KW) of the selected recombinants in the qKM4.08 region. Figure S4 (a) Expression analysis of the three genes contained in the final mapping region of qKM4.08. M indicates the molecular marker. (b) Amino acid sequence alignment of the AtVps29 and ZmVPS29 proteins. (c) Phylogenetic tree of ZmVPS29 and related proteins. Figure S5 Comparison of yield‐related traits in the T1/HZS‐F1 and corresponding BC2F1 populations. Figure S6 Transgenic validation of ZmVPS29 in the Zheng58 (Z58) background. Figure S7 Correlation between KM value and kernel yield per plant (YPP). Figure S8 Alignment of the nucleotide sequences of the ZmVPS29 coding region in HZS and LV28. Figure S9 Alignment of the nucleotide sequences of the ZmVPS29 promoter region in HZS and LV28. Figure S10 Comparison of 10‐KL (a) and 10‐KW (b) in the four haplotype groups. The values are presented as the means ± SDs. Figure S11 Expression of genes associated with auxin biosynthesis, transport, and degradation. Figure S12 A putative model depicting the role of ZmVPS29 in the regulation of kernel development in maize. Figure S13 Process for the fine mapping of qKM4.08. [file PBI-18-1004-s002.pdf]

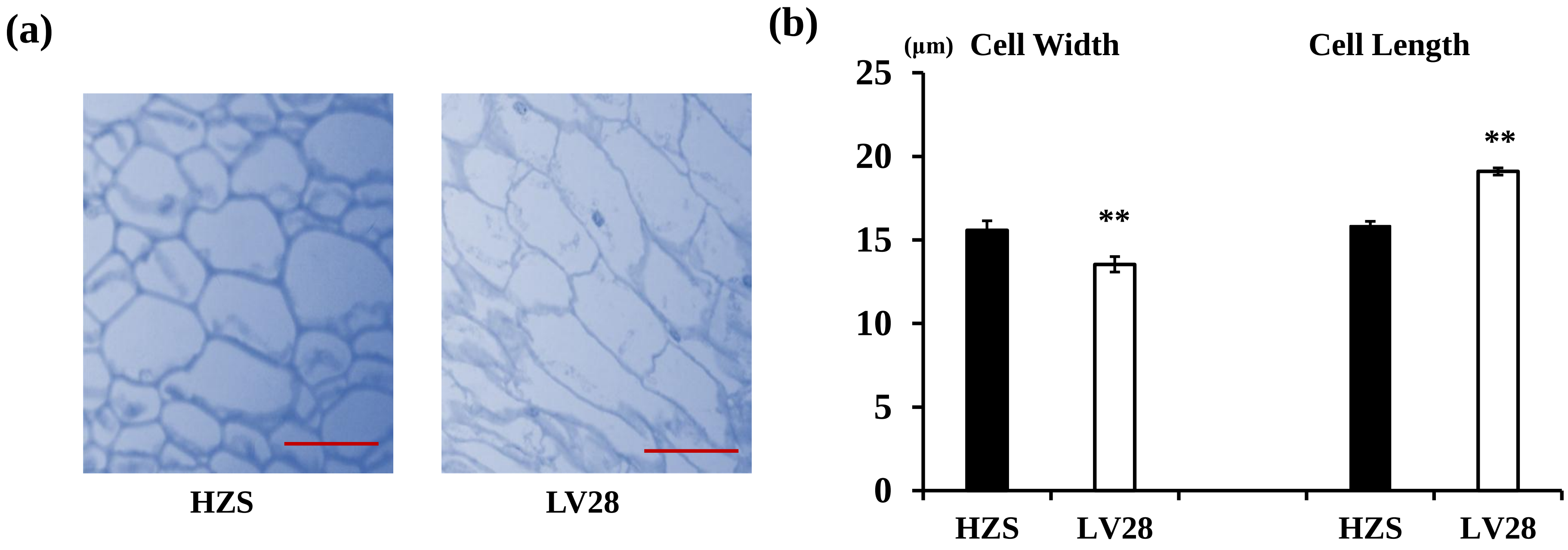

Figure S1 The morphology differences at 6 DAP between HZS and LV28 in the cytological level. (a) The scanning of seed coat of HZS and LV28 at 6 DAP. Bar = 20 μm. (b) The statistic analysis of seed coat between HZS and LV28. n =15, ** *P* < 0.01 (*t*-test)

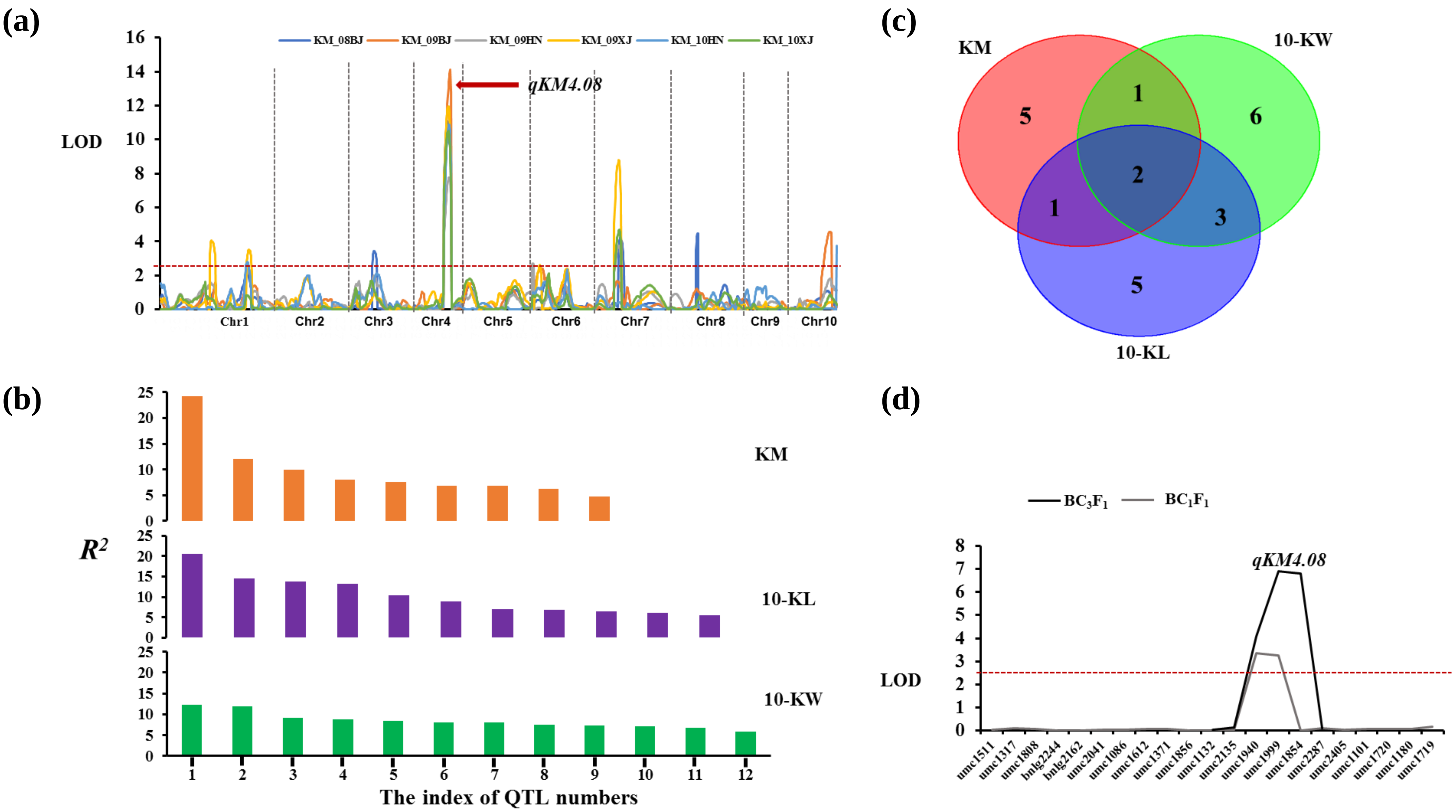

Figure S2 QTL analysis for KM. (a) QTL analysis for KM in the RIL population. (b) The phenotypic variation can be explained by the QTLs for 10-KL, 10-KW and KM in the RIL population. The x-axis indicates the number of QTLs, and the y-axis indicates the phenotypic variation explained by each QTL (R^2 %). (c) Number of overlapped QTLs for 10-KL, 10-KW and KM. (d) Validation of *qKM4.08* effects on KM in two backcross populations. *qKM4.08* was located in the umc1940-umc1987 interval (Chr4:220.65 Mb-231.90 Mb).

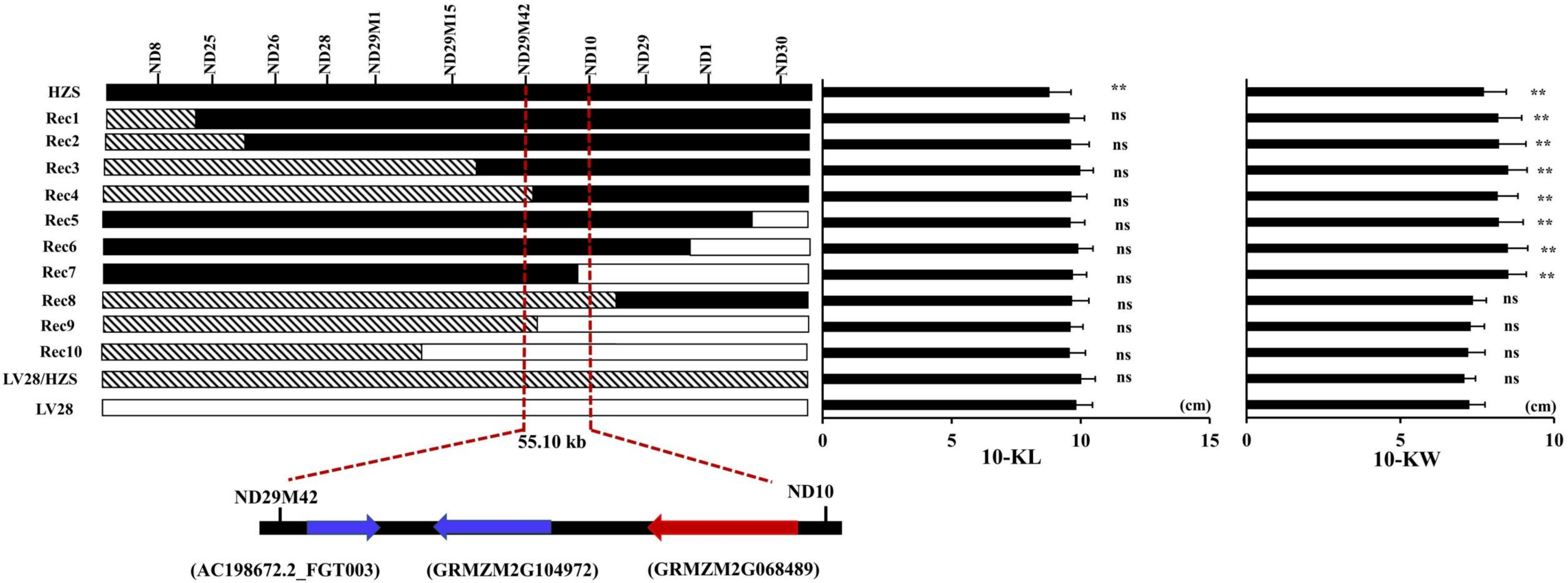

Figure S3 The genotypes and phenotypes (10-KL and 10-KW) of the recombinants in the *qKM4.08* region. ns means no significant difference, * $P < 0.05$, ** $P < 0.01$ (*t*-test).

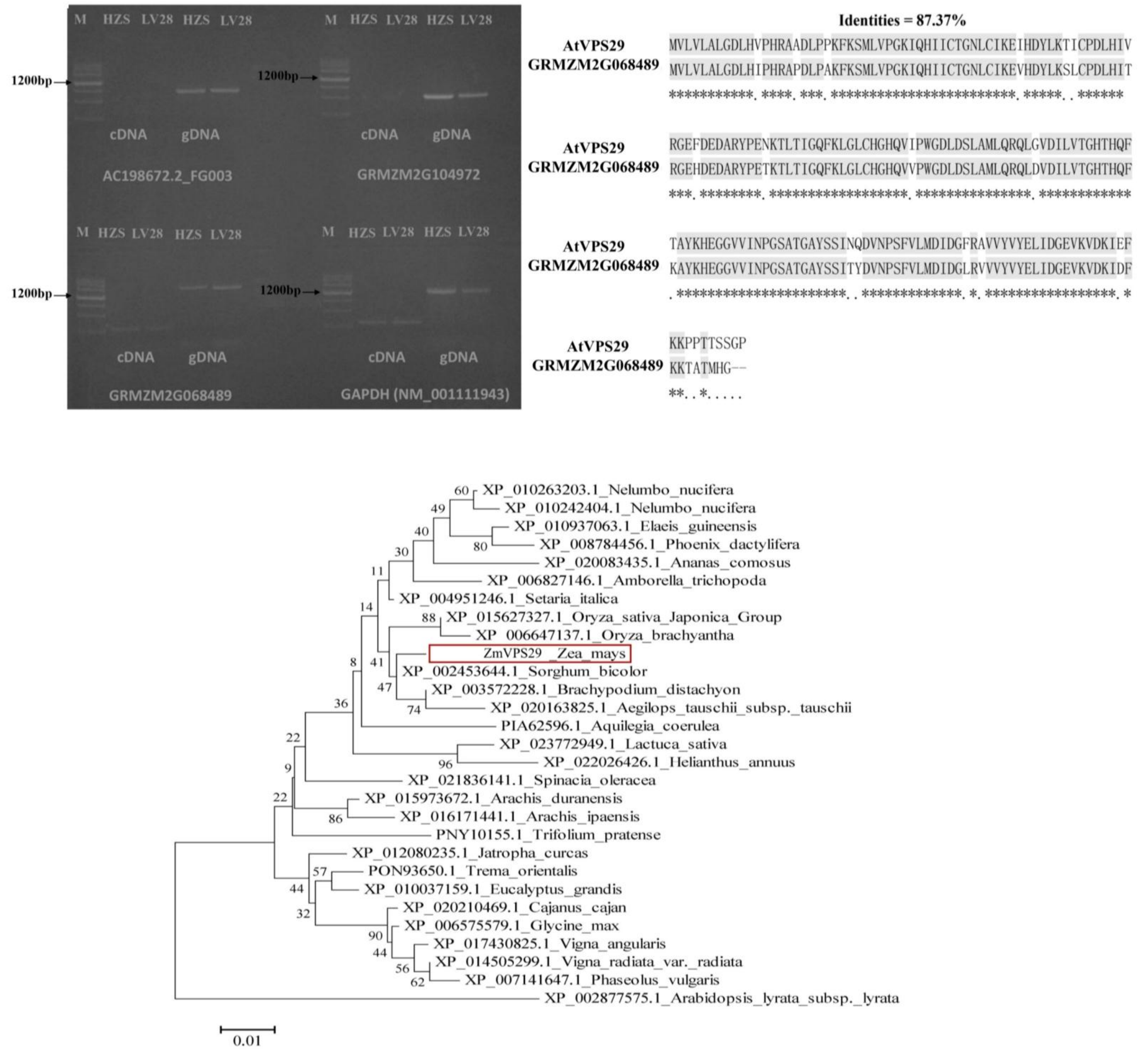

Figure S4 (a) Expression analysis of the three genes contained in the final mapping region of *qKM4.08*. M indicates the molecular marker. (b) Amino acid sequence alignment of AtVps29 and ZmVPS29 proteins. (c) Phylogenetic tree of ZmVPS29 and related proteins.

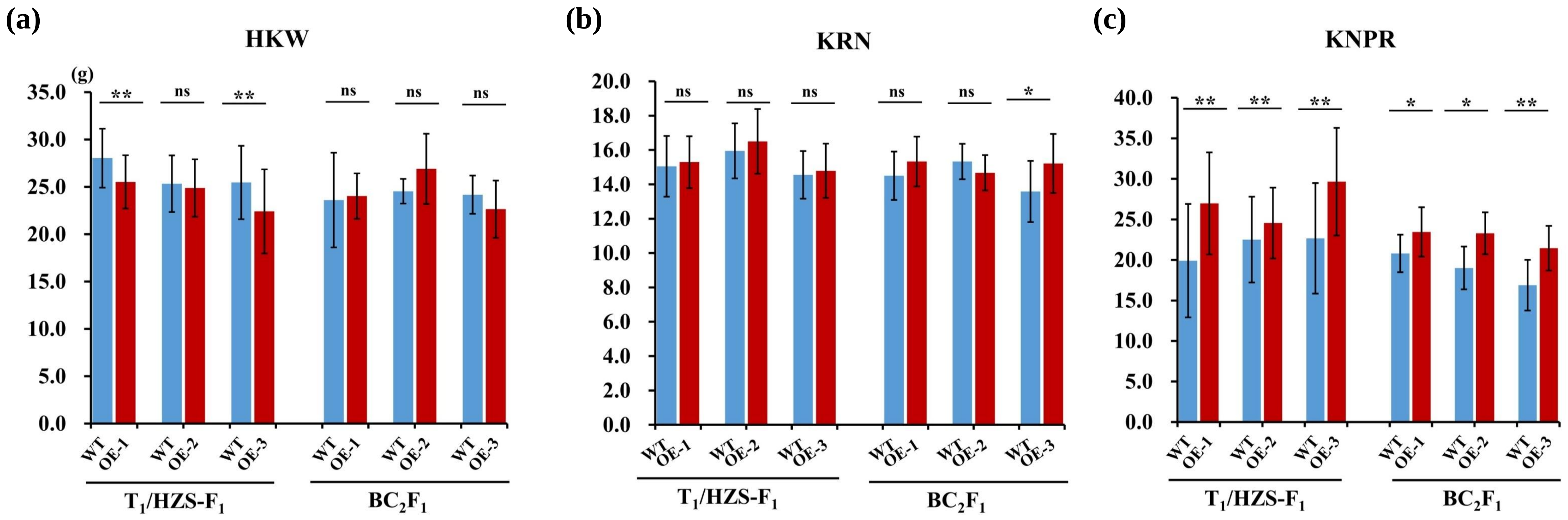

Figure S5 Comparison of yield-related traits in the T_1/HZS-F_1 and corresponding BC_2F_1 populations. (a-c) Comparison of HKW, kernel row number (KRN) and kernel number per row (KNPR). Values are the mean ± SE. ns means no significant difference, * $P < 0.05$, ** $P < 0.01$ (*t*-test).

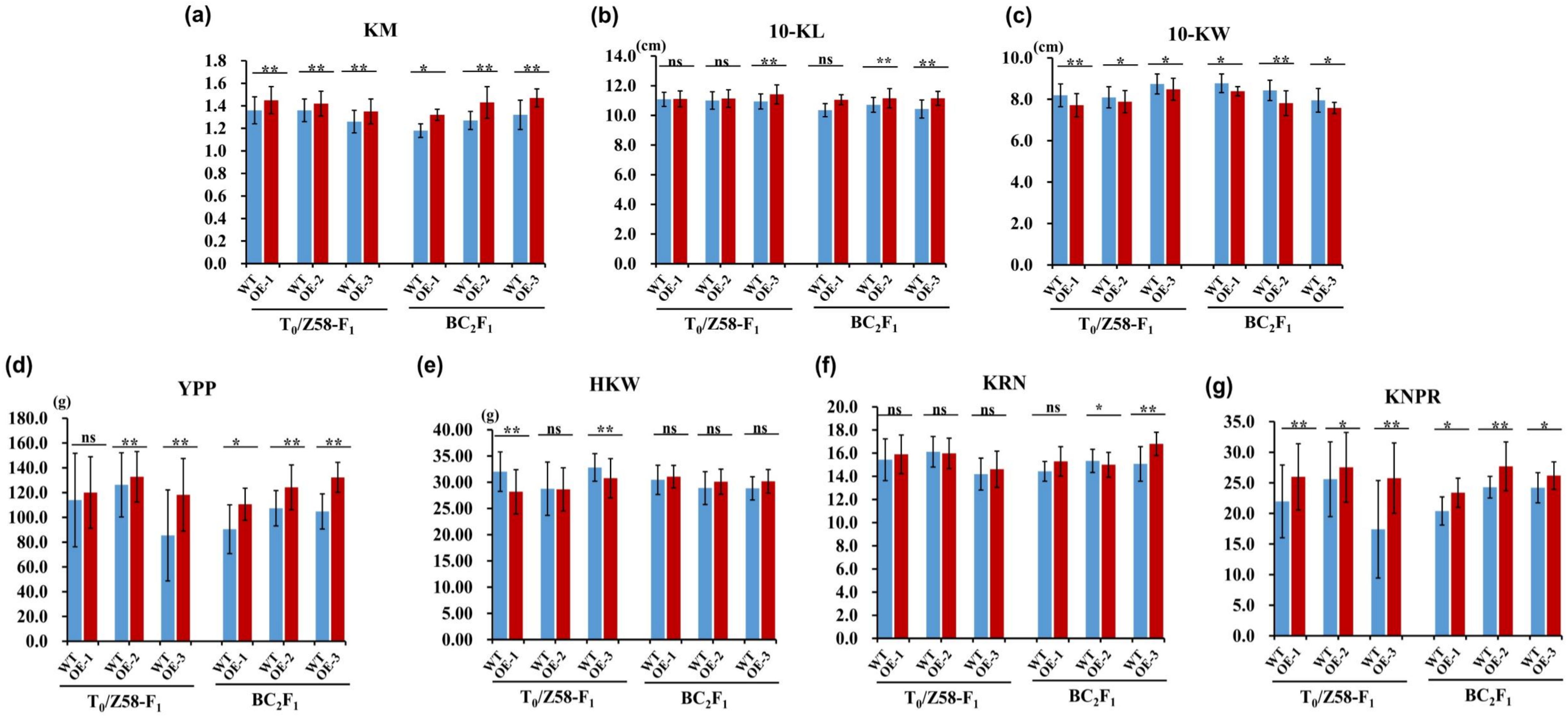

Figure S6 Transgenic validation of *ZmVPS29* in the Zheng58 (Z58) background. (a-g) Comparison of kernel size and other yield-related traits between the *ZmVPS29* overexpression transgenic plants and the non-transgenic plants in the T_1/Z58-F_1 and the corresponding BC_2F_1 populations (three independent transgenic events were used in this analysis). (a) KM (10-KL/10-KW). (b) 10-Kernel length (10-KL). (c) 10-Kernel width (10-KW). (d) Kernel yield per plant (YPP). (e) Hundred-kernel weight (HKW). (f) Kernel row number (KRN). (g) Kernel number per row (KNPR). Values are the mean ± SD. ns means no significant difference, * $P < 0.05$, ** $P < 0.01$ (*t*-test).

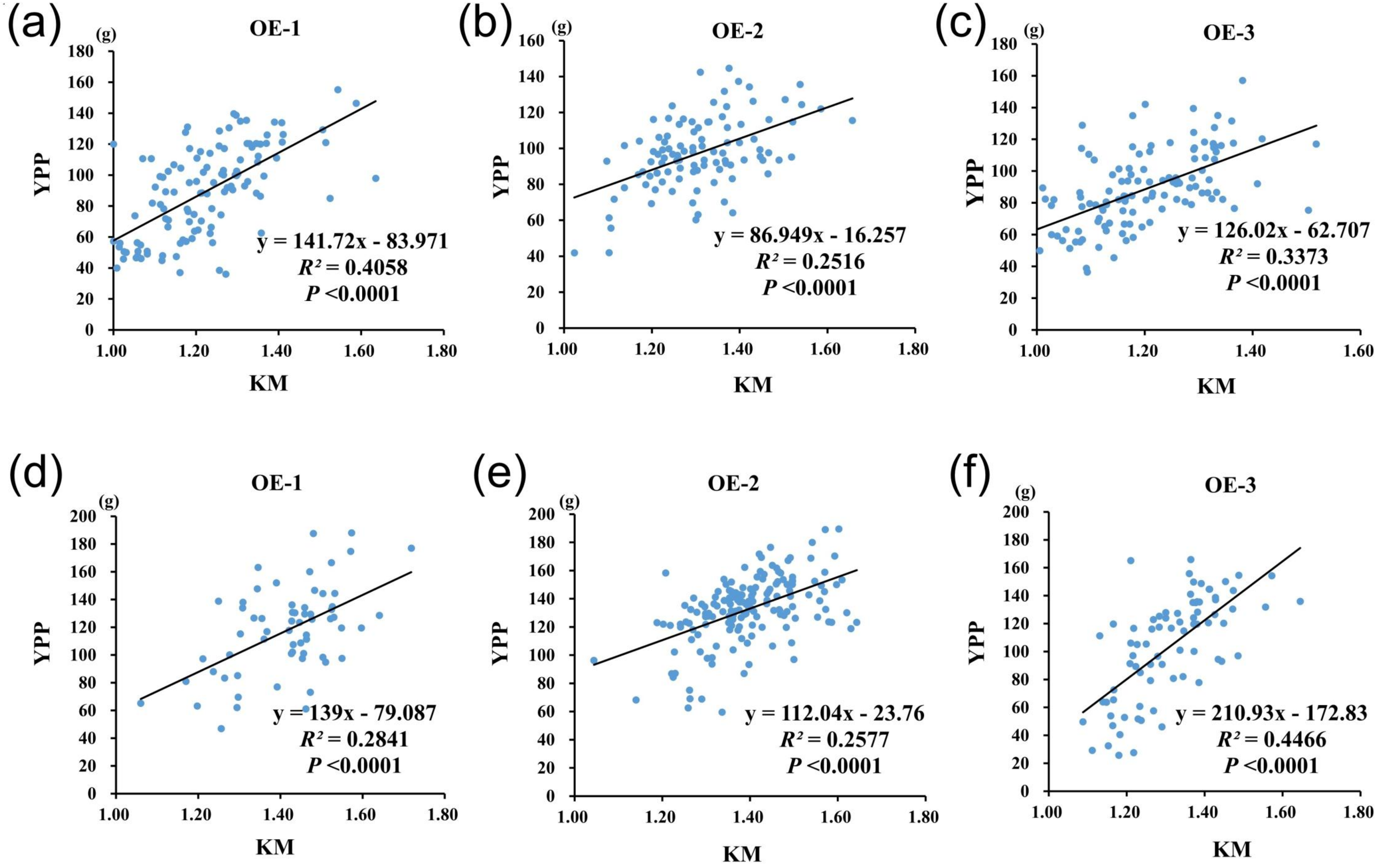

Figure S7 Correlation between KM value and kernel yield per plant (YPP). (a-c) Correlation between KM and YPP in plants from different transgenic events in the T_1/HZS-F_1 population. (a-f) Correlation between KM and YPP in plants from different transgenic events in the BC_2F_1 population in the HZS background.

```
ZmVPS29_B73     ATGGTGCTTGTGCTTGCGCTGGGGGATCTGCACATCCCGCACCGGGCGCCCGACCTCCCC
ZmVPS29_HZS     ATGGTGCTTGTGCTTGCGCTGGGGGATCTGCACATCCCGCACCGGGCGCCCGACCTCCCC
ZmVPS29_LV28    ATGGTGCTTGTGCTTGCGCTGGGGGATCTGCACATCCCGCACCGGGCGCCCGACCTCCCC
                ************************************************************
ZmVPS29_B73     GCCAAATTCAAGTCCATGCTCGTGCCCGGCAAGATCCAACACATCATCTGCACTGGCAAT
ZmVPS29_HZS     GCCAAATTCAAGTCCATGCTCGTGCCCGGCAAGATCCAACACATCATCTGCACTGGCAAT
ZmVPS29_LV28    GCCAAATTCAAGTCCATGCTCGTGCCCGGCAAGATCCAACACATCATCTGCACTGGCAAT
                ************************************************************
ZmVPS29_B73     CTCTGCATCAAGGAAGTCCATGACTACCTGAAAAGCCTTTGCCCTGATCTCCATATTACC
ZmVPS29_HZS     CTCTGCATCAAGGAAGTCCATGACTACCTGAAAAGCCTTTGCCCTGATCTCCATATTACC
ZmVPS29_LV28    CTCTGCATCAAGGAAGTCCATGACTACCTGAAAAGCCTTTGCCCTGATCTCCATATTACC
                ************************************************************
ZmVPS29_B73     AGAGGTGAACATGATGAGGATGCTCGATACCCAGAGACTAAGACACTTACAATTGGTCAG
ZmVPS29_HZS     AGAGGTGAACATGATGAGGATGCTCGATACCCAGAGACTAAGACACTTACAATTGGTCAG
ZmVPS29_LV28    AGAGGTGAACATGATGAGGATGCTCGATACCCAGAGACTAAGACACTTACAATTGGTCAG
                ************************************************************
ZmVPS29_B73     TTTAAGCTTGGGCTGTGCCATGGCCATCAGGTTGTTCCATGGGGCGACCTGGACTCCCTG
ZmVPS29_HZS     TTTAAGCTTGGGCTGTGCCATGGCCATCAGGTTGTTCCATGGGGCGACCTGGACTCCCTG
ZmVPS29_LV28    TTTAAGCTTGGGCTGTGCCATGGCCATCAGGTTGTTCCATGGGGCGACCTGGACTCCCTG
                ************************************************************
ZmVPS29_B73     GCGATGCTCCAGCGGCAGCTGGACGTGGACATCCTGGTGACCGGGCACACGCACCAGTTC
ZmVPS29_HZS     GCGATGCTCCAGCGGCAGCTGGACGTGGACATCCTGGTGACCGGGCACACGCACCAGTTC
ZmVPS29_LV28    GCGATGCTCCAGCGGCAGCTGGACGTGGACATCCTGGTGACCGGGCACACGCACCAGTTC
                ************************************************************
ZmVPS29_B73     AAGGCATATAAGCACGAGGGAGGCGTGGTGATCAACCCTGGCTCTGCCACGGGCGCCTAC
ZmVPS29_HZS     AAGGCATATAAGCACGAGGGAGGCGTGGTGATCAACCCTGGCTCTGCCACGGGCGCCTAC
ZmVPS29_LV28    AAGGCATATAAGCACGAGGGAGGCGTGGTGATCAACCCTGGCTCTGCCACGGGCGCCTAC
                ************************************************************
ZmVPS29_B73     AGCAGCATCACTTACGACGTGAACCCAAGCTTTGTGCTGATGGACATCGACGGGCTCCGT
ZmVPS29_HZS     AGCAGCATCACTTACGACGTGAACCCAAGCTTTGTGCTGATGGACATCGACGGGCTCCGT
ZmVPS29_LV28    AGCAGCATCACTTACGACGTGAACCCAAGCTTTGTGCTGATGGACATCGACGGGCTCCGT
                ************************************************************
ZmVPS29_B73     GTGGTGGTGTACGTCTACGAGCTGATTGACGGCGAGGTGAAGGTGGACAAAATCGACTTC
ZmVPS29_HZS     GTGGTGGTGTACGTCTACGAGCTGATTGACGGCGAGGTGAAGGTGGACAAAATCGACTTC
ZmVPS29_LV28    GTGGTGGTGTACGTCTACGAGCTGATTGACGGCGAGGTGAAGGTGGACAAAATCGACTTC
                ************************************************************
ZmVPS29_B73     AAGAAGACTGCGACGATGCACGGCTAG
ZmVPS29_HZS     AAGAAGACTGCGACGATGCACGGCTAG
ZmVPS29_LV28    AAGAAGACTGCGACGATGCACGGCTAG
                ***************************
```

Figure S8 Alignment of the nucleotide sequences of the coding region of *ZmVPS29* in HZS and LV28.

```
HZS_Promoter    TGCGATGTGTTCGTGAGAGGTCTAGGCCGTCGTCTCCTAGTCAACTTTGGGTTGCTGGAT
LV28_Promoter   TGCGATGTGTTCGTGAGAGGTCTAGGCCGTCGTCTCCTAGTCAACTTTGGGTTGATGGAT

HZS_Promoter    CGTTGTCTCCTTACCATGTAATTATTTATTTATTTTGTACAGAACTCCTATTATATAGTA
LV28_Promoter   CGTTGTCTCCTTACCATGTATTTATTTATTTATTTTGTACAGAACTCCTATTATATAGTA
                   AuxRE
HZS_Promoter    AAGTTATTACATTCATTTCTGTACCATGATTTATCATATGTGTGAGACTTGGTCCCAGCA
LV28_Promoter   AAGTTATTACATTCGTTTCTGTACCATGATTTATCATATGTGTGAGACTTGGTCCCAGCG

HZS_Promoter    CACCTGGTGATTATGTTCGCGCCTGGGTCCCTAAAACTCGAGTGTGACAGAGGGCGTGCG
LV28_Promoter   CACCTGGTGATTATGTTCGCGCCCGGGTCCCTAAAACCCGAGTGTGACAGAGGGCGTGCG

HZS_Promoter    GAGATGGCCGGAAAACGCGTGAACGTGGACGCATCCACGATGGGGGCGTGGGTGGGAGGT
LV28_Promoter   GAGATGGCCGGAAAACGCGTGAACGTGGACGCATCCACGATGGGGGCGTGGGTGGGAGGT

HZS_Promoter    TAGGGACGAGGTCTGACGGGTGGGTCCGCAGGACAGAGAGAGAGGATGAGCGCGTGCGAG
LV28_Promoter   TAGGGACGAGGTCTGACGGGTGGGTCCGCAGGGCAGAGAGAGAGGATGAGCGCGTGCGAG

HZS_Promoter    GGGATCAGCACCGACAGGCCGACCCCACAGAGCACAGAGAGAGAGAGA--AGGGGTGCGT
LV28_Promoter   GGGATCAGCACCGACAGGCCGACCCCACAGAGCAGAGAGAGAGAGAGAGAAGGGGTGCGT

HZS_Promoter    GGGCTGGCGCCGATAGGCCGGGTCCGTCTGTCCGACTTGGGCTGAAATGGTTTTTTCTAT
LV28_Promoter   GGGCTGGCGCCGATAGGCCGGGTCCGTCTGTCCGACTTGGGCTGAAATGGTTTTTTCTAT
                                              AuxRE
HZS_Promoter    TTTTCAGGGAATTTCTAGTGTTTTTCTATTTATGTTCTCTAGGGTTTTGAATTCAAATTC
LV28_Promoter   TTTTCAGGGAATTTCTAATGTTTTTCTATTTATGTTCTCTAGGGTTTTGAATTCAAATTC

HZS_Promoter    AAACTAAATCAAACATGTGCAACAATTTAAAAAATATTTGGAGCTCAGCACGATGCAACA
LV28_Promoter   AAACTAAATCAAACATGTGCAACAATTTAAAAAATATTTGGAGCTCACCACGATGCAACA

HZS_Promoter    TTTCATGACTCATATTATTTTGACAAAATAAAATAATCAACCCCTCACTAATTAAGCTAA
LV28_Promoter   TTTCATGACTCATATTGTTTTGACAAAATAAAATAATCAACCCCTCACTAATTAAGCTAA

HZS_Promoter    TTCTACTAAAAAGAAAAAGAGAGAGAAACTAGAGAGAGAGGAGTAACACCTGAATTTGGT
LV28_Promoter   TTCTACTAAAAAGAAAAAGAGAGAGAAACTAGAGAGAGAGGAGTAACACCTGAATTTGGT

HZS_Promoter    AGGTAATTAGAAAGAAATTTTATACCCCCAAATTTAGGGTGTTACAACCTCTGCGGACAA
LV28_Promoter   AGGTAATTAGAAAGAAATTTTATACCCCCAAATTTAGGGTGTTACAACCTCTGCGGACAA

HZS_Promoter    GGCAAAATGCGCAGTTATTTGAAAGGAATATTCGAACCAAGGTTCGATCTACCACGGCCA
LV28_Promoter   GGCAAAATGCGCAGTTATTTGAAAGGAATATTCGAACCAAGGTTCGATCTACCACGGCCA

HZS_Promoter    CGGCCCGCCGGCGGCGGCGGCACGCGCGTGTGTGGTCGTCTTTCATTCTTTTCCCAACTT
LV28_Promoter   CGGCCCGCCGGCGGCGGCGGCACGCGCGCGTGTGGTCGTTTTTCATTCTTTTCCCAACTT

HZS_Promoter    CTTAATAGATGCACCAATTGGTGCACCTATTTAAGTTGATTGATTGATCTCTTAAACTTA
LV28_Promoter   CTTAATAGATGCACCAATTGGTGCACCTATTTAAGTTGATTGATTGATCTCTTGAACTTA

HZS_Promoter    CAATATGGTACTAAATTATTAGTACACCATATCATTAAAGTGGACCACTAGCATTGACTA
LV28_Promoter   CAATATGATACTAAATTATTAGTATACCATATCATTAAAGTGGACCACTAGCATTGACTA

HZS_Promoter    TTATTGAATATTAATTGGGCCAAGCCAACATTAATCCAATATAAACAATGATAATTAGGT
LV28_Promoter   TTATTGAATATTAATTGGGCCAAGCCAACATTAATCCAATATAAACAATGATAATTAGGT

HZS_Promoter    AATATTTTGAATAATATGGATGACATAAATCTTGAAAATATAGGATACATGGAGATTATG
LV28_Promoter   AATATTTTGAATAATATGGATGGCATAAATCTTGAAAATATAGGATACATGGAGATTATG

HZS_Promoter    TATTGAACTTGAGAAATCTATAGACAGAGTTTCTGAATTGAACTAGGTAAATCTGTAAAC
LV28_Promoter   TATTGAACTTGAGAAATCTATAGACAGAGTTTCTGAATTGAACTAGGTAAATCTGTAAAC

HZS_Promoter    GGGAATACTGAATTATACTCCCTCTCTTTAGAAAATTAACAAATTCTTACAATACTTGAT
LV28_Promoter   GGGAATACTGAATTATACTCCCTCTCTTTAGAAAATTAACAAATTCTTACAATACTTGAT

HZS_Promoter    GTATGTATTATATATATGTGTATAGATTTATTATCATTCATTTGAATATAGACATAAAA-
LV28_Promoter   GTATGTATTATATATATGTGTATAGATTTATTATCATTCATTTGAATATAGACATAAAAA

HZS_Promoter    CCAAGATCTAAAACGAATACTATTTTAGACGGAGAGAGTATAGATTTGTAAGAATCTATT
LV28_Promoter   CCAAGATCTAAAACGAATACTATTTTAGACGGAGAGAGTATAGATTTGTAAGAATCTATT

HZS_Promoter    TAGCTGATGTATCCTTTCAGTTAGGATTCAATTTTTTTTTAATAGGAGGATTCAAATTTT
LV28_Promoter   TAGCTGATGTATCCTTTCAGTTAGGATTCAATTTTTTTTTAATAGGAGGATTCAAATTTT

HZS_Promoter    TTGATAGTACATCTAGATTCTAGACCGTCGATCTTTCAAACGTTGAAAAGAGGAAGAAGA
LV28_Promoter   TTGATAGTACATCTAGATTCTAGACCGTCGATCTTTCAAACGTTGAAAAGAGGAAGAAGA

HZS_Promoter    GCTTGACGTTTTGCTTAGCTTAACGCTAGCCCAACGAACTTGGCGAAACCGATGGAGCCT
LV28_Promoter   GCTTGACGTTTTGCTTAGCTTAACGCTAGCCCAACGAACTTGGCGAAACCGATGGAGCCT

HZS_Promoter    CAAACGGGCCGTATCCGTCGTGCACAGCATGCTAAAGCCCAGTGCTGACGACGACGAACA
LV28_Promoter   CAAACGGGCCGTATCCGTCGTGCACAGCATGCTAAAGCCCAGTGCTGACGACGACGAACA

HZS_Promoter    ACACTCATCCGGCCCGGTCATGAGCCCAATGCGGGGCCACTTCGTCGTTTTTGACAAGTA
LV28_Promoter   ACACTCATCCGGCCCGGTCATGAGCCCAATGCGGGGCCACTTCGTCGTTTTTGACAAGTA

HZS_Promoter    GACCCACAGTGGCCCCACGTGCTTGCCCCTCCCTTTCTCTTCTCCCCTTGTGTCGCGCTC
LV28_Promoter   GACCCACAGTGGCCCCACGTGCTTGCCCCTCCCTTTCTCTTCTCCCCTTGTGTCGCGCTC
                                                                  AuxRE
HZS_Promoter    GCGCATCTGAAGCAAGGGGGGGAAGGGCCAAGGGGGATCGGTCTTGTTCTCGGAGAGGGT
LV28_Promoter   GCGCATCTGAAGCAAGGGGGGGAAGGGCCAAGGGGGATCGGTCTTGTTCTCGGAGAGGGT

HZS_Promoter    TGAGCTGCTTGACGGTTTTGACTCGGATCATGGTGTGCAGCTCTATCAGATAGAGAGCTG
LV28_Promoter   TGAGCTGCTTGACGGTTTTGACTCGGATCATGGTGTGCAGCTCTATCAGATAGAGAGCTG

HZS_Promoter    ACGTGAGGTGTGAGACGCGGATCGTGGAGTACTACTCAGTAGGAGA-------------G
LV28_Promoter   ACGTGAGGTGTGAGACGCGGATCGTGGAGTACTACTCAGTAGGAGATCAAGAGGAAGGAG

HZS_Promoter    GTGGGGAGGGGGAAGAAGGCGATAGCCGGGGAGGatg···
LV28_Promoter   GTGGGGAGGGGGAAGAAGGCGATAGCCGGGGAGGatg···
```

Figure S9 Alignment of the nucleotide sequences of the promoter region of *ZmVPS29* in HZS and LV28.

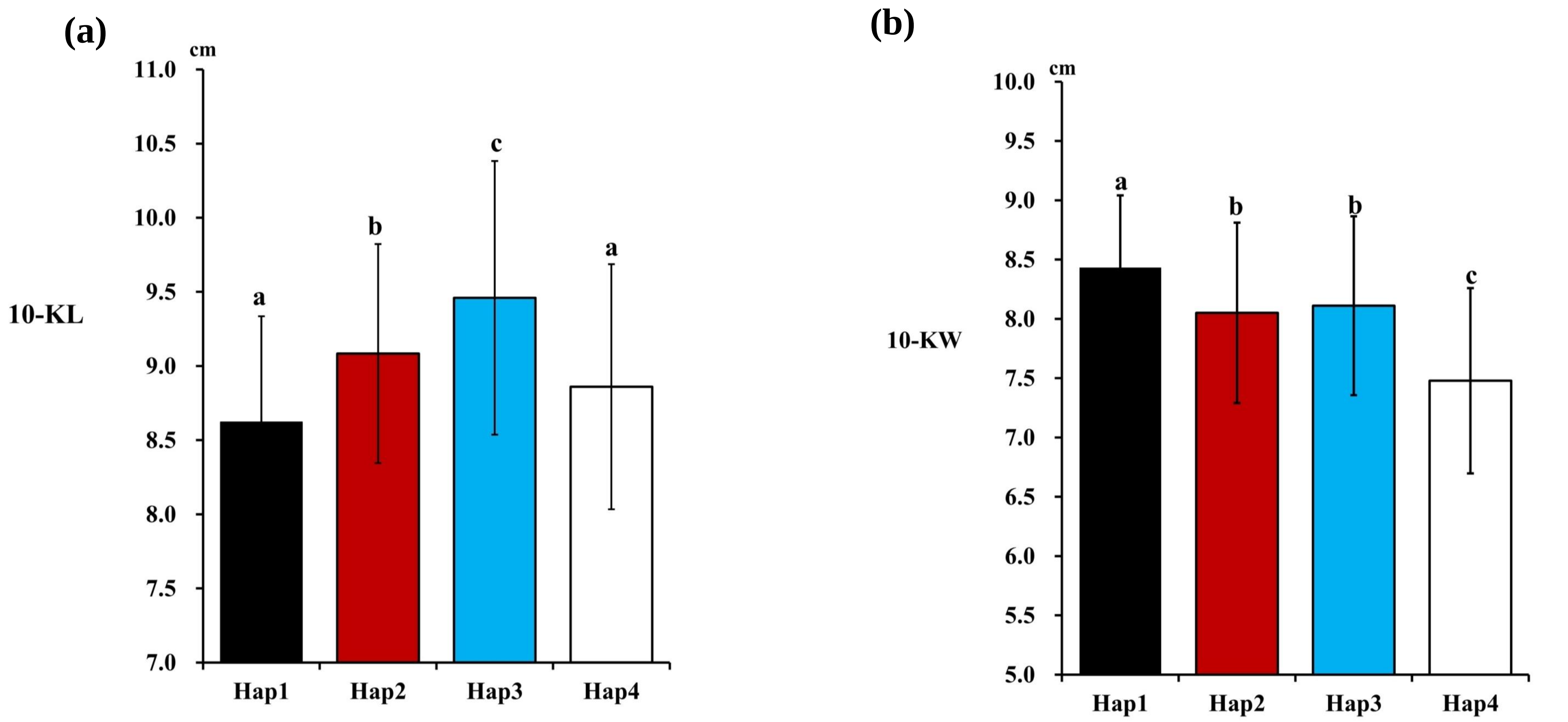

Figure S10 Comparison of 10-KL (a) and 10-KW (b) in the four haplotype groups. Values are the mean ± SD.

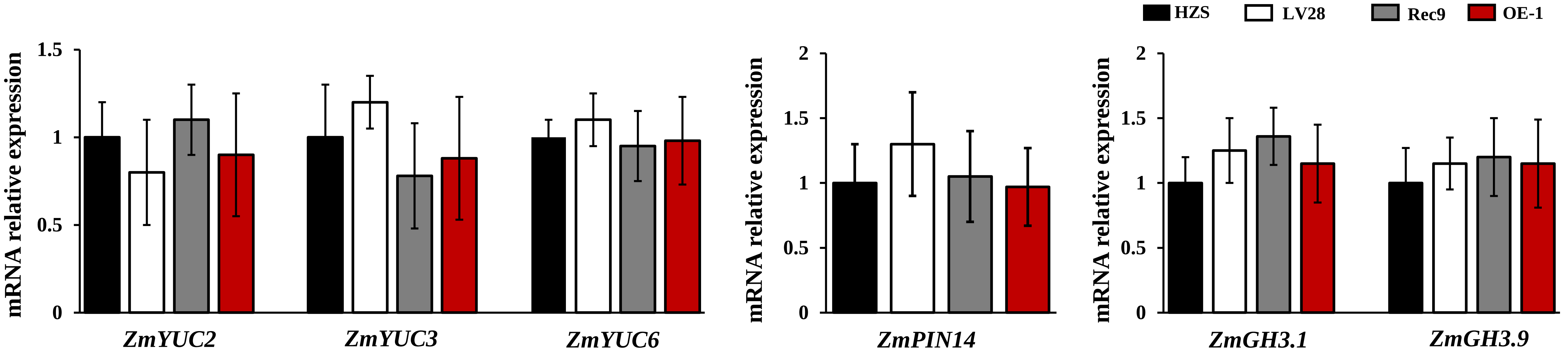

Figure S11 Expression of genes for auxin biosynthesis, transport, and degradation.

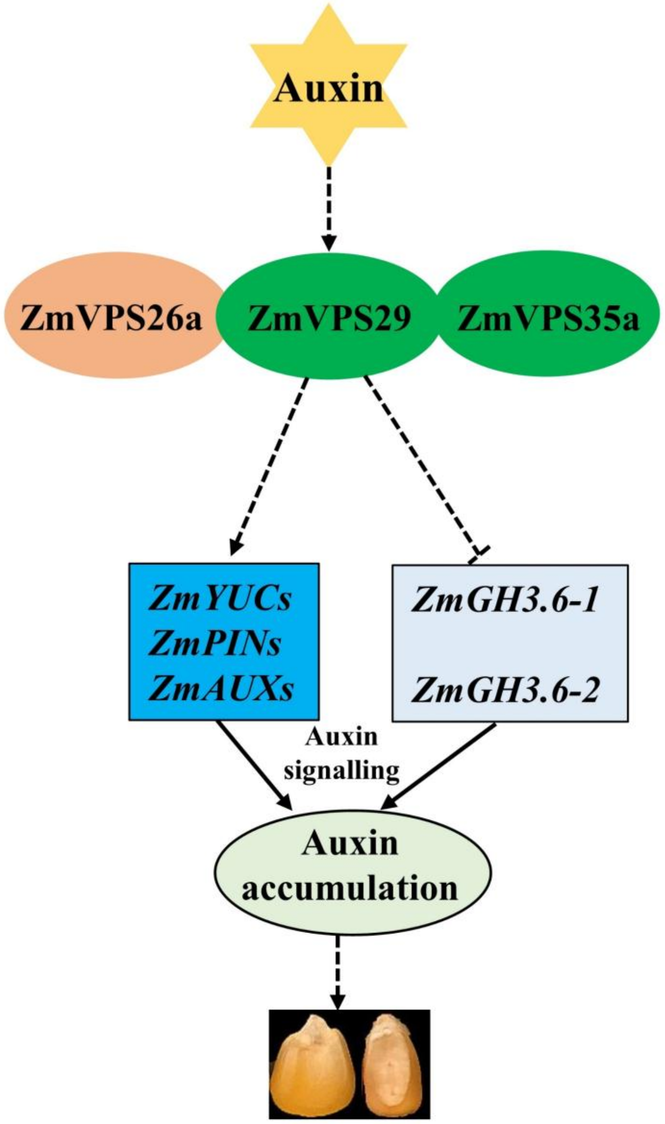

Figure S12 A putative model depicting the role of ZmVPS29 in the regulation of kernel development in maize.

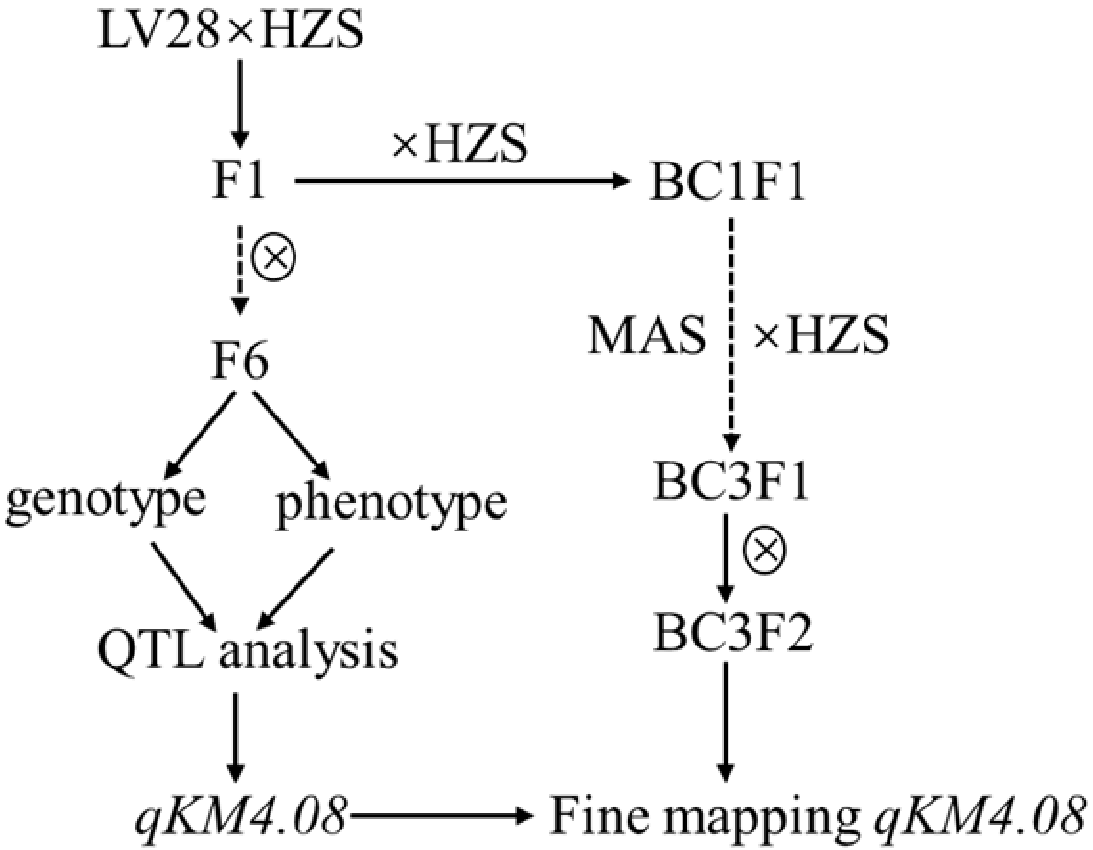

Figure S13 The process for fine mapping *qKM4.08*
